# Study on Mechanical Properties and Crack Evolution of Basalt Fiber-Reinforced Desert Sand High-Strength Concrete Based on DIC

**DOI:** 10.3390/ma19163486

**Published:** 2026-08-18

**Authors:** Pengyu Wang, Qiaoxia An, Lingyan Xu, Junwen Wan, Rui Yin

**Affiliations:** School of Water Conservancy and Architectural Engineering, Tarim University, Alar 843300, China; 13614166885@163.com (P.W.);

**Keywords:** desert sand, high-strength concrete, basalt fiber, digital image correlation, crack evolution, microscopic mechanism

## Abstract

This study investigates the strength development, crack evolution and toughening mechanism of basalt fiber-reinforced desert sand high-strength concrete. An L9(3^3^) orthogonal design was first used to optimize the reference mixture, after which basalt fibers with volume fractions of 0, 0.3%, 0.4% and 0.5% were incorporated. Mechanical testing, digital image correlation, SEM, XRD, TG and FTIR were combined to clarify the relationship among fiber dosage, crack propagation and microstructural reinforcement mechanisms. The optimized matrix mixture was obtained with a water-to-binder ratio of 0.32, a desert sand replacement ratio of 40% and a fly ash content of 20%. The incorporation of basalt fiber had little influence on the 28 d compressive strength, whereas the splitting tensile strength was markedly improved. The highest splitting tensile strength was observed in the 0.4% fiber group, reaching 5.46 MPa, which was 12.81% higher than that of the reference mixture. DIC results showed that basalt fiber reduced strain localization and limited crack opening. The 0.5% group had the lowest COD, while the 0.4% group showed a better balance among tensile strength, strain redistribution and crack-opening control. SEM observations showed fiber bridging and fiber–matrix interaction near the fracture region. Meanwhile, XRD, TG-DTG and FTIR showed no obvious changes in the main phases or functional groups, indicating that the improvement was mainly related to the physical crack-control effect of basalt fibers rather than chemical modification of the matrix. Overall, 0.4% basalt fiber was identified as the preferred dosage for the present system.

## 1. Introduction

Concrete serves as a fundamental material in a broad range of infrastructure projects. With the development of bridges, high-rise buildings, heavy-load structures and structures exposed to complex environments, concrete materials are required to possess higher strength, durability and long-term service performance. Owing to its superior compressive performance, compact microstructure and relatively low water-to-binder ratio, high-strength concrete can provide enhanced load-bearing capacity, reduce member cross-sections and lower self-weight. Therefore, developing high-strength concrete that combines high strength, crack resistance and resource-utilization characteristics contributes to better structural performance while supporting greener and lower-carbon concrete construction [1]. Meanwhile, the sustained large-scale exploitation of natural river sand has led to resource depletion, ecological degradation and rising transportation costs. Developing local alternative fine aggregates has therefore become an important approach to promoting greener and more regionalized development of the concrete industry [2]. Desert sand reserves in southern Xinjiang, China, are abundant, and incorporating desert sand into high-strength concrete can reduce reliance on natural river sand and improve the utilization efficiency of local resources [3].

High-strength concrete usually improves matrix compactness and durability by reducing the water-to-binder ratio, optimizing the composition of cementitious materials and improving aggregate gradation. Fly ash, slag and silica fume are commonly incorporated as supplementary cementitious constituents to refine the pore structure through physical filling and secondary pozzolanic reactions, which have important influences on later-age strength development and durability improvement [4]. Against the background of shortage of natural river sand resources, desert sand or aeolian sand has been gradually used in the research of high-strength concrete and ultra-high performance concrete. When incorporated at a suitable dosage, desert sand can supplement the fine particle fraction, improve the aggregate packing state, and affect the workability, mechanical properties, the pore system and the properties of the interfacial transition zone [5,6]. Zhang et al. reported that high-performance concrete in which aeolian sand completely replaces conventional fine aggregate is generally designed with a relatively low water-to-binder ratio and a high binder dosage, but its volume stability and shrinkage-cracking issues still need attention [7]; Zhu et al. further demonstrated that rational mixture proportioning, optimization of the binder system, and fiber reinforcement can enhance the fresh-state performance, mechanical strength and long-term durability of concrete produced entirely with aeolian sand [8]. Zaitri et al.’s study investigating high-performance concrete incorporating ground dune sand also showed that fine particulate materials such as desert sand have certain application potential in high-performance concrete [9]. Therefore, desert sand functions as more than a direct substitute for natural river sand in high-strength concrete, but rather it jointly affects material properties together with the water-to-binder ratio, cementitious material composition, pore structure and interfacial bonding state. At present, related studies are still mostly focused on mix proportion, strength and durability evaluation, while insufficient attention has been paid to crack initiation, propagation and localized failure behavior during loading.

Despite its high compressive capacity and compact hardened structure, high-strength concrete often exhibits pronounced brittleness owing to the combined effects of a low water-to-binder ratio and a high-strength cementitious matrix [1,4]. During loading, once microcracks initiate at the aggregate-paste interface or local defects, they tend to propagate rapidly and form a main crack, leading to a rapid decline in post-peak bearing capacity. For desert sand high-strength concrete, desert sand fine particles can, to a certain extent, fill the inter-aggregate pores and improve matrix compactness, but its grading characteristics, specific surface area and interfacial bonding state also affect the initiation location and propagation path of cracks [7,8]. Consequently, conventional strength parameters, including compressive and splitting tensile strengths, cannot adequately characterize the progressive evolution from localized damage to global failure in desert sand high-strength concrete.

Discrete fibers are commonly used to improve crack resistance and deformation capacity in high-strength concrete. In desert sand concrete, steel, polypropylene, glass and basalt fibers have been used to enhance tensile or flexural performance and control crack propagation [10,11]. Basalt fiber has high tensile strength and elastic modulus and is compatible with inorganic cementitious matrices. However, its resistance to the alkaline pore solution of cement-based materials should be interpreted cautiously, because it depends on fiber composition, surface coating or sizing, matrix alkalinity and exposure conditions [12,13].

Basalt-based reinforcement systems have also shown potential in different building applications. For example, coated basalt textile-reinforced inorganic systems have been used for masonry strengthening because of their light weight, mineral-substrate compatibility and convenient application [14]. Previous studies have shown that basalt fiber can improve tensile strength, flexural capacity, toughness and crack-control performance, while its effect depends strongly on fiber dosage and dispersion quality [12,13,15,16]. Therefore, basalt fiber was selected in this study as a crack-control reinforcement for desert sand high-strength concrete.

Traditional mechanical tests can obtain macroscopic indicators such as compressive strength and splitting tensile strength, but they have difficulty reflecting the whole process from crack initiation to propagation and eventually through-failure instability. DIC technology has the advantages of non-contact and full-field measurement, and has been used for crack width identification, crack propagation monitoring and quantitative analysis of crack opening displacement in concrete [17,18,19,20]. At the same time, a suite of microstructural characterization techniques, including SEM, XRD, TG-DTG and FTIR, can help explain the macroscopic performance and crack-evolution results from the perspectives of the fiber–matrix interface, main phases, thermal decomposition behavior and functional-group structures [21,22]. At present, studies on desert sand high-strength concrete are mostly focused on material composition, mechanical properties and durability evaluation, while research that combines reference mix proportion optimization, basalt fiber reinforcement, full-process DIC analysis under compression and splitting tensile loading, and microscopic characterization is still relatively limited. For this reason, this paper takes desert sand high-strength concrete in southern Xinjiang as the research object, determines the reference mix proportion through an L9(3^3^) orthogonal test, and sets basalt fiber volume fractions of 0%, 0.3%, 0.4% and 0.5%, conducting mechanical property tests, DIC crack evolution analysis and microstructure analysis, with emphasis on revealing the effects of basalt fiber on its strength development, strain localization, crack opening displacement and microscopic crack-resistance mechanism.

## 2. Test Scheme and Design

### 2.1. Test Raw Materials

The cementitious materials used in this test were Conch brand P·O 42.5 ordinary Portland cement, supplied by Xinjiang Suyuan Packaging Technology Co., Ltd. (Changji, China), and Class I fly ash, supplied by Longze Water Purification Materials Co., Ltd. (Gongyi, China). The 3-day and 28-day compressive strengths of the cement were 26.7 MPa and 49.4 MPa, respectively; the fly ash had a 45 μm square-hole sieve residue of 8.5%, a water demand ratio of 94% and a loss on ignition of 3.34%. The coarse aggregate consisted of two size fractions of crushed stone, 5–10 mm and 10–20 mm, which were mixed at a mass ratio of 4:6. The fine aggregate was composed of natural river sand and desert sand from the southern Xinjiang region, where the desert sand replaced the natural river sand according to the designed replacement ratio. The reinforcing material was 12 mm chopped basalt fiber supplied by Shanghai Chenqi Chemical Technology Co., Ltd. (Shanghai, China), with a density of 2.69 g/cm^3^, tensile strength above 2000 MPa, elastic modulus above 85 GPa, diameter of approximately 17 μm and elongation at break of 2.5%. The alkaline durability of basalt fibers depends on fiber composition, surface treatment, matrix alkalinity and exposure conditions [12,13]. Since this study mainly focuses on mechanical properties and crack evolution, alkaline durability was not specifically investigated. The supplier also did not provide coating or sizing information; therefore, their effects on alkaline durability were not directly evaluated and are discussed only with reference to previous studies. The superplasticizer was a polycarboxylate high-performance water-reducing agent supplied by Shanghai Chenqi Chemical Technology Co., Ltd. (Shanghai, China), with a water-reducing rate of approximately 25%, and the mixing water was tap water from Aral City, Xinjiang, China.

**Figure 1:**
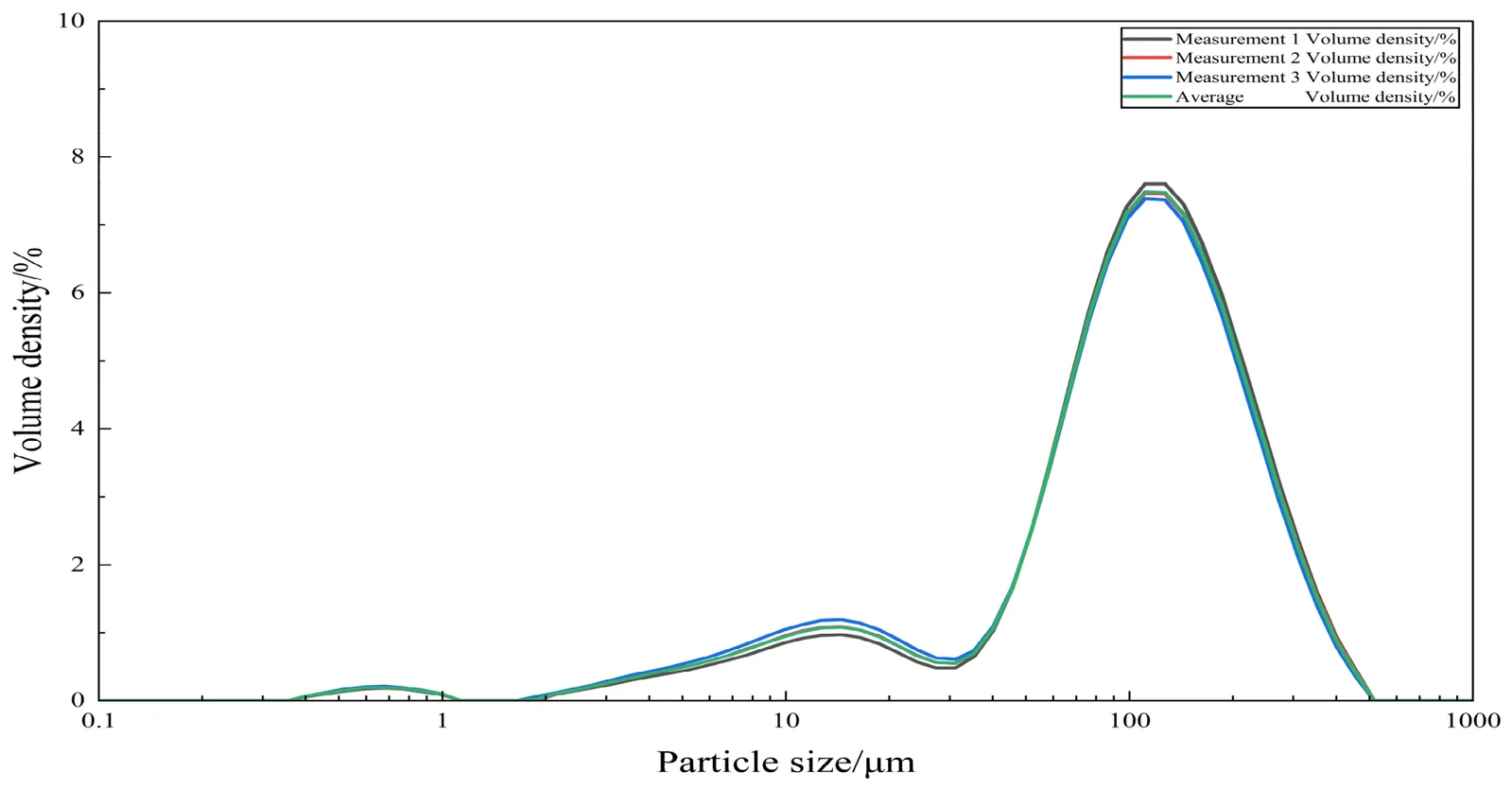
Particle size distribution curve of desert sand.

**Figure 2:**
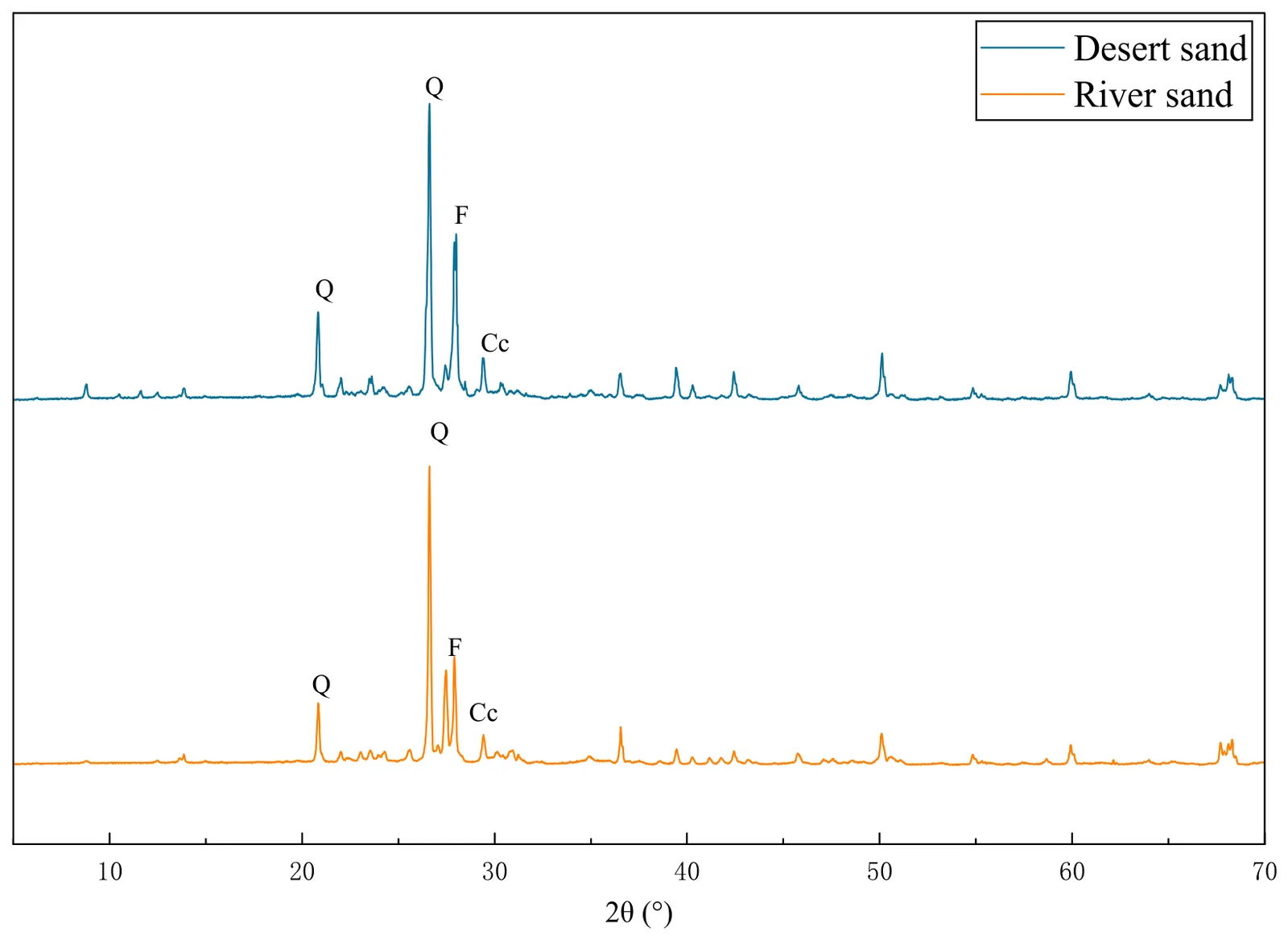
XRD patterns of desert sand and river sand. Q: quartz; F: feldspar; Cc: calcite.

**Figure 3:**
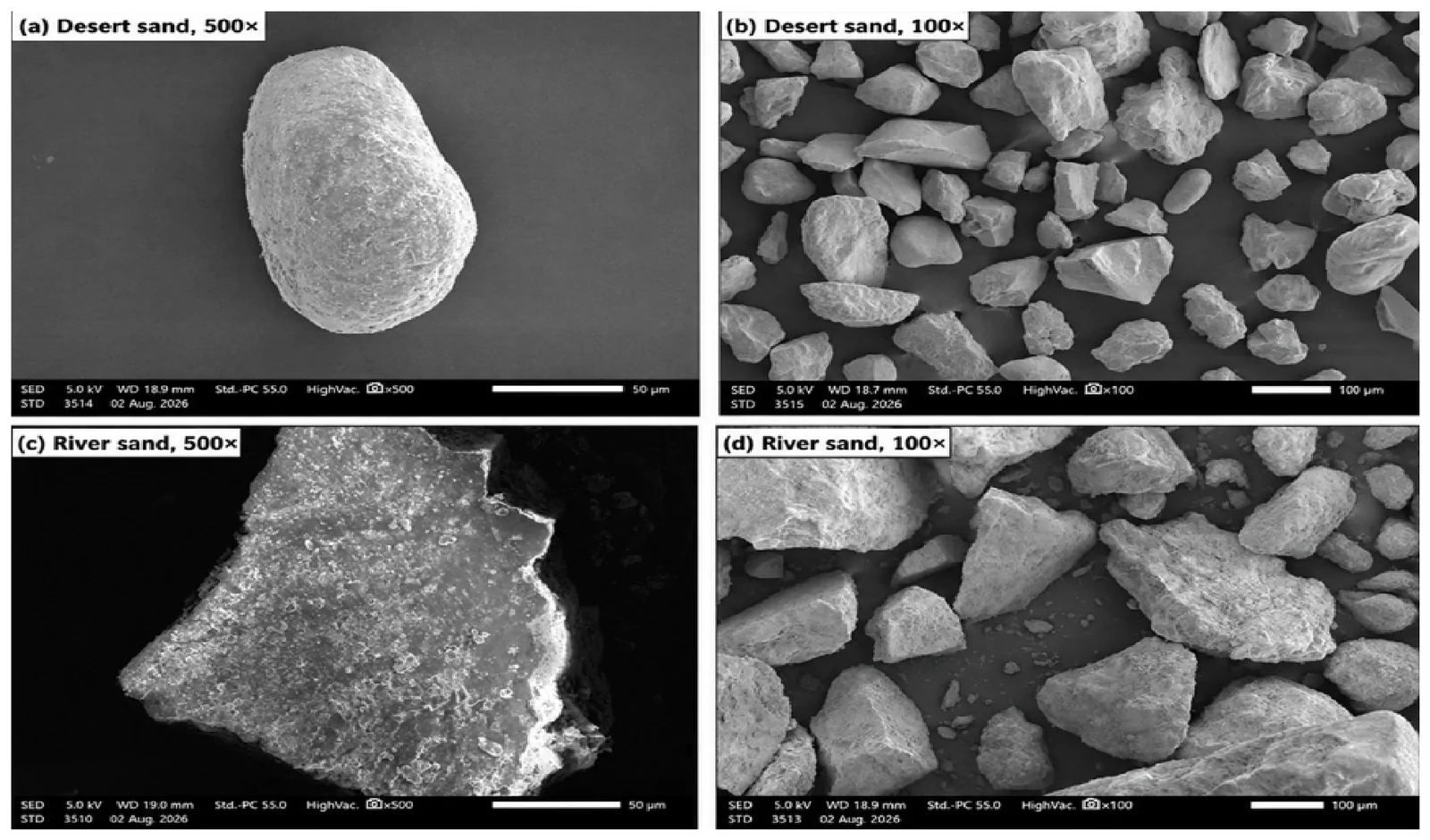
SEM images showing the particle morphology of desert sand and river sand: (**a**) desert sand, 500×; (**b**) desert sand, 100×; (**c**) river sand, 500×; (**d**) river sand, 100×.

The chemical composition of desert sand was determined using a ZSX Primus III+ wavelength-dispersive X-ray fluorescence spectrometer (Rigaku Corporation, Akishima, Tokyo, Japan), operated at 50 kV and 60 mA. Its particle-size distribution was measured using a Mastersizer 3000 laser diffraction particle size analyzer (Malvern Panalytical Ltd., Malvern, UK). The chemical composition, physical properties, particle-size distribution, mineralogical composition and particle morphology of the fine aggregates are shown in Table 1 and Table 2 and Figure 1, Figure 2 and Figure 3. As shown in Table 1, the desert sand mainly consists of SiO_2_, CaO and Al_2_O_3_, with contents of 50.35%, 25.28% and 11.56%, respectively, indicating siliceous–calcareous characteristics.

Table 2 shows that river sand and desert sand have similar apparent densities of 2635 and 2625 kg/m^3^, respectively, while their water absorption values are 1.80% and 0.44%, respectively. In contrast, their fineness moduli differ considerably. The fineness modulus of desert sand is 0.214, much lower than that of river sand (2.54), indicating its much finer particle size. This is consistent with the particle-size distribution in Figure 1, where the desert sand shows a unimodal distribution with a main peak at 100–150 μm. The D10, D50 and D90 values are 15.6, 115 and 257 μm, respectively.

To identify the mineralogical origin of the high CaO content, XRD analysis was performed on both sands. As shown in Figure 2, both samples are mainly composed of quartz, with typical peaks at approximately 2θ=20.8°, 26.6°, 36.5°, 50.1° and 59.9°. Feldspar-related peaks are observed near 27.4°–28.0°. Compared with river sand, desert sand shows a clearer calcite peak near 29.4°, indicating that its relatively high CaO content is mainly related to Ca-bearing carbonate minerals, especially calcite, rather than free CaO.

Figure 3 compares the particle morphology of the two sands. Desert sand particles are finer, and some grains are rounded or sub-rounded with relatively smooth surfaces. River sand particles are coarser and more angular, with rougher surfaces and sharper edges. These differences suggest that desert sand mainly provides a fine-filling effect, whereas river sand contributes more to skeleton support and mechanical interlocking. Therefore, proper desert sand replacement may improve particle packing, while excessive replacement may reduce workability because of the increased specific surface area.

### 2.2. Mix Proportion and Test Design

To obtain a reference mixture design for desert sand high-strength concrete suitable for the subsequent basalt fiber reinforcement tests, this paper adopts an L9(3^3^) orthogonal test for pre-optimization of the reference mix proportion. In the orthogonal test, the water-to-binder ratio is set to 0.32, 0.34 and 0.36, the desert sand replacement rate is set to 30%, 40% and 50%, and the fly ash content is set to 0%, 10% and 20%. The constituent quantities for each mixture are presented in Table 3.

To ensure reproducibility of the orthogonal optimization, the evaluation procedure was clarified. Slump, 28 d compressive strength and 28 d splitting tensile strength were normalized using the maximum-value method:

(1) $$R_{ij}=\frac{X_{ij}}{X_{j,max}}$$

where $R_{ij}$ is the normalized value of the j-th indicator for the i-th mixture, $X_{ij}$ is the measured value, and $X_{j,max}$ is the maximum value of that indicator among the nine mixtures. Since no segregation was observed, all three indicators were treated as positive indicators. The weights assigned to slump, compressive strength and splitting tensile strength were 0.20, 0.45 and 0.35, respectively. The comprehensive score was calculated as:

(2) $$S_{i}=0.20R_{i,s}+0.45R_{i,c}+0.35R_{i,t}$$

where $S_{i}$ is the comprehensive score, and $R_{i,s}$, $R_{i,c}$ and $R_{i,t}$ are the normalized slump, 28 d compressive strength and 28 d splitting tensile strength, respectively.

Based on the range analysis, A1B2C3 was identified as the predicted preferred combination, corresponding to a water-to-binder ratio of 0.32, a desert sand replacement ratio of 40% and a fly ash content of 20%. Because this combination was not included in the original L9(3^3^) design, a confirmation test was conducted. The verified mixture was designated as DSHC-JZ and used as the reference matrix for the subsequent basalt fiber tests, in which only the fiber volume fraction was varied at 0%, 0.3%, 0.4% and 0.5%.

Considering that basalt fiber may reduce the workability of fresh concrete, the superplasticizer dosage was increased with increasing fiber content to maintain acceptable casting performance. The complete mix proportions of DSHC-JZ and the fiber-reinforced mixtures are listed in Table 4, including the basalt fiber mass, actual superplasticizer dosage and slump values.

The superplasticizer dosage was adjusted to maintain acceptable casting performance; therefore, the slump values reflect the combined influence of fiber incorporation and admixture dosage.

### 2.3. Specimen Preparation and Test Methods

**Figure 4:**
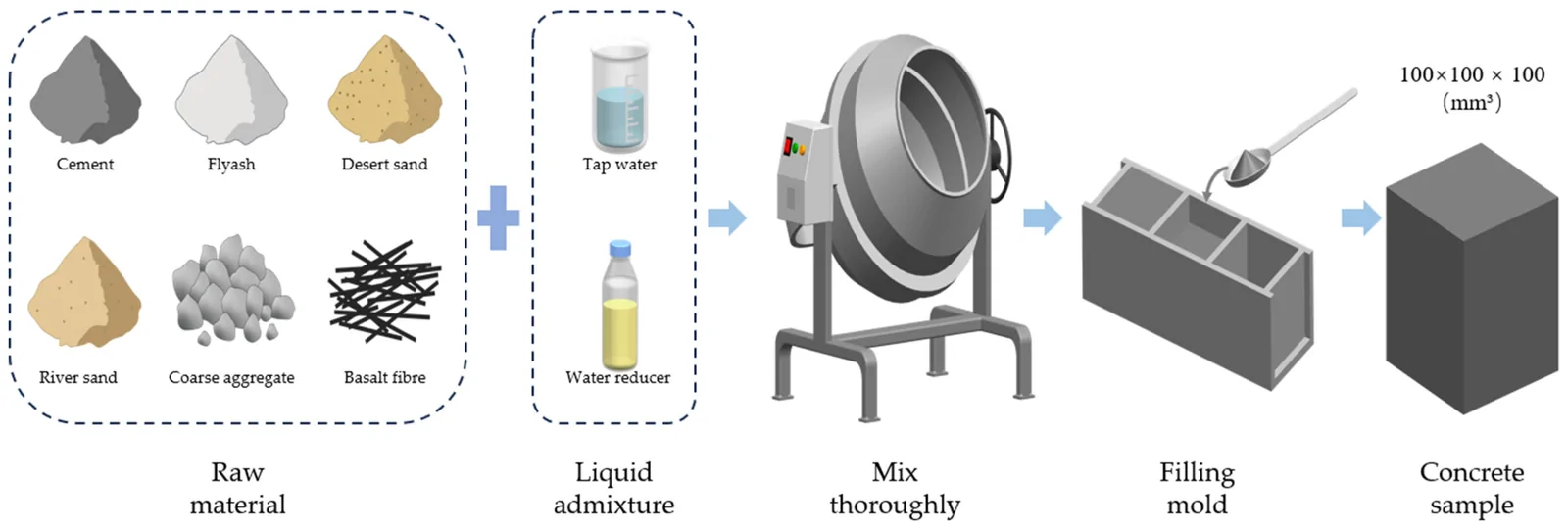
Flow chart of specimen preparation.

All constituents, including cement, fly ash, fine and coarse aggregates, basalt fiber, water and superplasticizer, were batched in accordance with the specified mixture proportions. The coarse aggregate, river sand and desert sand were initially dry-blended for 2 min, after which cement and fly ash were introduced and mixing was continued for an additional 2 min; the basalt fiber was added by sprinkling in small amounts at multiple times to reduce fiber agglomeration; finally, the water and superplasticizer mixture was added and mixing continued for 3 min. After the mixture was placed into molds and vibrated, it was demolded after standing for 24 h and then placed in a standard curing room with a temperature of (20 ± 2) °C and a relative humidity of not less than 95% for curing until the specified age. The specimen preparation procedure is illustrated in Figure 4.

#### 2.3.1. Slump Test

The slump test was conducted in accordance with JGJ/T 281-2012 Technical Specification for Application of High-Strength Concrete [23]. Each group of mixture was tested three times, and the average value was taken as the slump result of that group.

#### 2.3.2. Compressive Strength Test

According to the Standard Test Methods for Physical and Mechanical Properties of Concrete (GB/T 50081-2019) [24], cubic specimens measuring 100 mm on each side were adopted for the test, with no fewer than three parallel specimens in each group. The compressive strength test was performed using an HCT306A microcomputer-controlled automatic compression testing machine (WANCE Technologies Ltd., Shenzhen, China) with a maximum capacity of 3000 kN, under load control at a loading rate of 0.5 MPa/s. The mean result from the parallel specimens was reported as the compressive strength for the corresponding mixture.

#### 2.3.3. Splitting Tensile Strength Test

According to the Standard Test Methods for Physical and Mechanical Properties of Concrete (GB/T 50081-2019) [24], splitting tensile jigs and load transmission devices were used in conjunction during the test. In this study, splitting tensile tests were conducted on 100 mm concrete cubes. The splitting tensile test was performed under load control at 0.05 MPa/s. Three replicate specimens were tested for each mixture, and the mean result was reported as its splitting tensile strength. Mechanical test results were expressed as mean ± standard deviation. Welch ANOVA with Games–Howell post-hoc tests was used for the 28 d strength comparisons, and the individual values and statistical results are given in Tables S1–S3.

### 2.4. DIC Test Method

**Figure 5:**
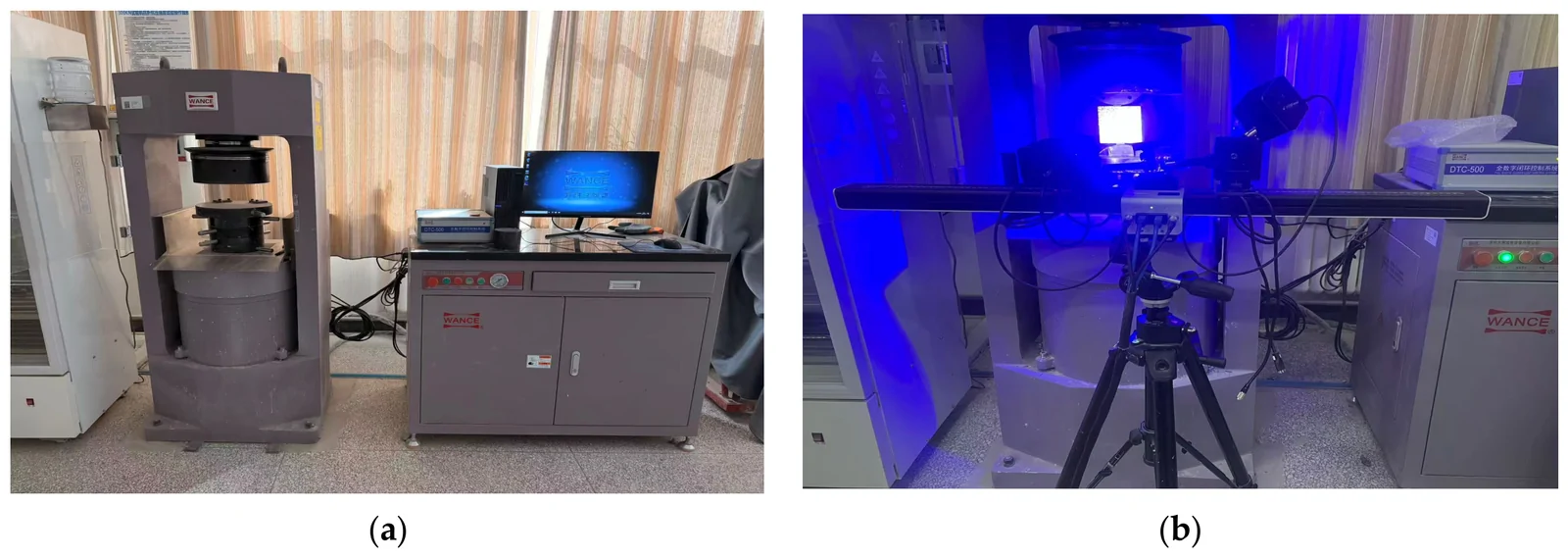
Mechanical property test equipment and digital image correlation (DIC) test system: (**a**) Microcomputer-controlled fully automatic compression testing machine; (**b**) DIC test apparatus.

To reveal the deformation localization and crack evolution laws of basalt fiber-reinforced desert sand high-strength concrete during compression and splitting tensile processes, this paper adopts Digital Image Correlation (DIC) technology for non-contact monitoring of full-field displacement and strain on the specimen surface [17], as shown in Figure 5b. A two-dimensional DIC measurement system was used in the test, mainly consisting of an industrial camera, a 50 mm lens and a constant cold light source. During the test, the camera was arranged facing the observation surface of the specimen, and the distance between the camera and the specimen surface was approximately 500 mm, to reduce imaging distortion and ensure that the observation area completely covered the main crack development range of the specimen.

Before formal loading, speckle preparation was performed on the observation surface of the specimen. First, a white matte primer was uniformly sprayed on the specimen surface, and after it dried, black speckles were randomly marked to form a random speckle field with relatively high grayscale contrast and uniform distribution. During loading, DIC image acquisition was synchronized with the compression testing machine, with an image acquisition interval of 400 ms/frame, and the load data at the corresponding moments were recorded simultaneously. At the adopted quasi-static loading rates, the 400 ms interval, equivalent to 2.5 frames/s, corresponded to stress increments of about 0.20 MPa in compression and 0.02 MPa in splitting tension, which was adequate for comparing the main pre-peak strain-localization and crack-opening processes among mixtures. After the test, the acquired image sequences were processed using XTDIC deformation and strain measurement software (Version 9.7.2, XTOP 3D Technology (Xi’an) Co., Ltd., Xi’an, China) to obtain the displacement and maximum principal strain fields on the specimen surface.

Table 5 lists the main image acquisition and DIC calculation parameters. The image resolution was 5120 × 5120 pixels and the calibrated observation length was 85 mm, corresponding to 0.0166 mm/pixel. All specimens were processed using the same DIC parameters.

The image and load data were matched according to the synchronized acquisition sequence. Since the synchronization accuracy and measurement uncertainty were not independently calibrated, the DIC data were mainly used for comparative analysis. As a two-dimensional DIC system was used, out-of-plane displacement was not directly corrected, but its influence was minimized by perpendicular camera alignment and a fixed camera–light–specimen configuration. Therefore, the DIC results were interpreted mainly to compare in-plane strain localization and crack-opening behavior among different mixtures.

DIC monitoring was performed on selected representative specimens. Based on the mechanical results and typical failure modes, three mixtures, DSHC-JZ, DSHC-BF0.4 and DSHC-BF0.5, were selected for DIC analysis. For each mixture, one specimen was monitored during compression and one during splitting tensile loading; therefore, six specimens were monitored in total. The selected specimens had strength values close to the corresponding group mean and showed typical failure patterns.

The strain fields and COD curves presented in this study were obtained from representative specimens rather than averaged replicate DIC measurements. Therefore, the DIC-derived strain fields, strain concentration coefficients and COD values were mainly used to explain crack-evolution characteristics and to support the mechanical test results.

Four load levels, $P/P_{max}=0.3$, 0.6, 0.9 and 1.0, were selected to compare the maximum principal strain fields of different specimens. Here, P is the real-time load and $P_{max}$ is the peak load. For splitting tensile specimens, the main crack was identified from the maximum principal strain field, and COD was extracted using fixed virtual gauge-point pairs across the strain-localization band. Three gauge pairs were placed at y/H=0.75, 0.50 and 0.25. The initial gauge length $L_{g}$ was 10 mm, with the two points located approximately 5 mm from the crack on each side. The average COD was calculated from the three gauge pairs and used to evaluate crack opening and propagation stability.

### 2.5. Microscopic Test Methods

The microscopic tests included XRD, TG-DTG, FTIR and SEM. XRD analysis was performed using an Ultima IV X-ray diffractometer (Rigaku Corporation, Akishima, Tokyo, Japan) with Cu Kα radiation (λ = 1.54 Å), operated at 40 kV and 40 mA, to identify the main phases of the hardened matrix. TG-DTG analysis was conducted using a thermogravimetric analyzer (NETZSCH-Gerätebau GmbH, Selb, Germany), with a test temperature range from room temperature to 1000 °C and a heating rate of 10 °C/min under an air atmosphere, to analyze the thermal decomposition characteristics of bound water, Ca(OH)_2_ and CaCO_3_. FTIR analysis was performed using a Nicolet iS5 Fourier transform infrared spectrometer (Thermo Fisher Scientific, Waltham, MA, USA) over the range of 4000–400 cm^−1^ at a resolution of 4 cm^−1^. Each spectrum was collected using 32 scans with air as the background. SEM observations were performed using an Apreo scanning electron microscope (Thermo Fisher Scientific, Waltham, MA, USA) to observe fracture morphology, fiber bridging and fiber–matrix interfacial characteristics. Samples were taken from the fracture areas after compressive or splitting failure, with emphasis on the areas near the main crack, the basalt fiber–matrix interface and the desert sand–paste interface.

## 3. Results Analysis and Discussion

### 3.1. Pre-Optimization of Reference Mix Proportion

To obtain the reference mix proportion of desert sand high-strength concrete required for the subsequent basalt fiber reinforcement tests, this paper adopts the L9(3^3^) orthogonal test for pre-optimization of the water-to-binder ratio, desert sand replacement rate and fly ash content. The comprehensive performance evaluation was based on the slump, 28 d compressive strength and 28 d splitting tensile strength. Considering the different dimensions of each indicator, the indicators were first normalized, and then the comprehensive scores were calculated according to the weights of workability and mechanical properties. The corresponding results are presented in Table 6.

As shown in Table 6, the slump, 28 d compressive strength and 28 d splitting tensile strength of the nine mixtures ranged from 155 to 211 mm, 59.60 to 70.30 MPa and 3.34 to 4.10 MPa, respectively. These differences indicate that the water-to-binder ratio, desert sand replacement ratio and fly ash content affected the workability and mechanical properties of desert sand high-strength concrete. Among the nine tested mixtures, DSHC-2 achieved the highest comprehensive score of 0.953 and was therefore regarded as the best-performing tested mixture. To further identify the preferred level of each factor, range analysis was conducted using the comprehensive score as the evaluation indicator, and the results are shown in Table 7.

The range analysis in Table 7 shows that the desert sand replacement ratio had the greatest influence on the comprehensive score, followed by the water-to-binder ratio, whereas the fly ash content had a relatively small effect. The level-average scores of C2 and C3 were very close, with values of 0.8910 and 0.8913, respectively, which became identical after rounding to three decimal places. This indicates that the difference between 10% and 20% fly ash was limited in the present test range.

Based on the level-average results, A1B2C3 was identified as the predicted preferred combination, corresponding to a water-to-binder ratio of 0.32, a desert sand replacement ratio of 40% and a fly ash content of 20%. From a material perspective, an appropriate amount of desert sand can improve fine aggregate gradation and provide a micro-filler effect [25], while a lower water-to-binder ratio improves matrix compactness. Fly ash can also refine the paste structure through particle filling and later-age pozzolanic reactions [22,26].

Since A1B2C3 was not included in the original L9(3^3^) array, it was not directly used as the reference matrix based only on range analysis. An additional confirmation test was therefore conducted, and the results are shown in Table 8.

As shown in Table 8, the confirmation mixture DSHC-JZ showed a slump of 184 mm, a 28 d compressive strength of 70.60 MPa and a 28 d splitting tensile strength of 4.84 MPa. Compared with DSHC-2, DSHC-JZ maintained comparable compressive strength and showed higher slump and splitting tensile strength. Therefore, A1B2C3 was adopted as the verified reference matrix for the subsequent basalt fiber-reinforced mixtures.

### 3.2. Effect of Basalt Fiber on Mechanical Properties

**Figure 6:**
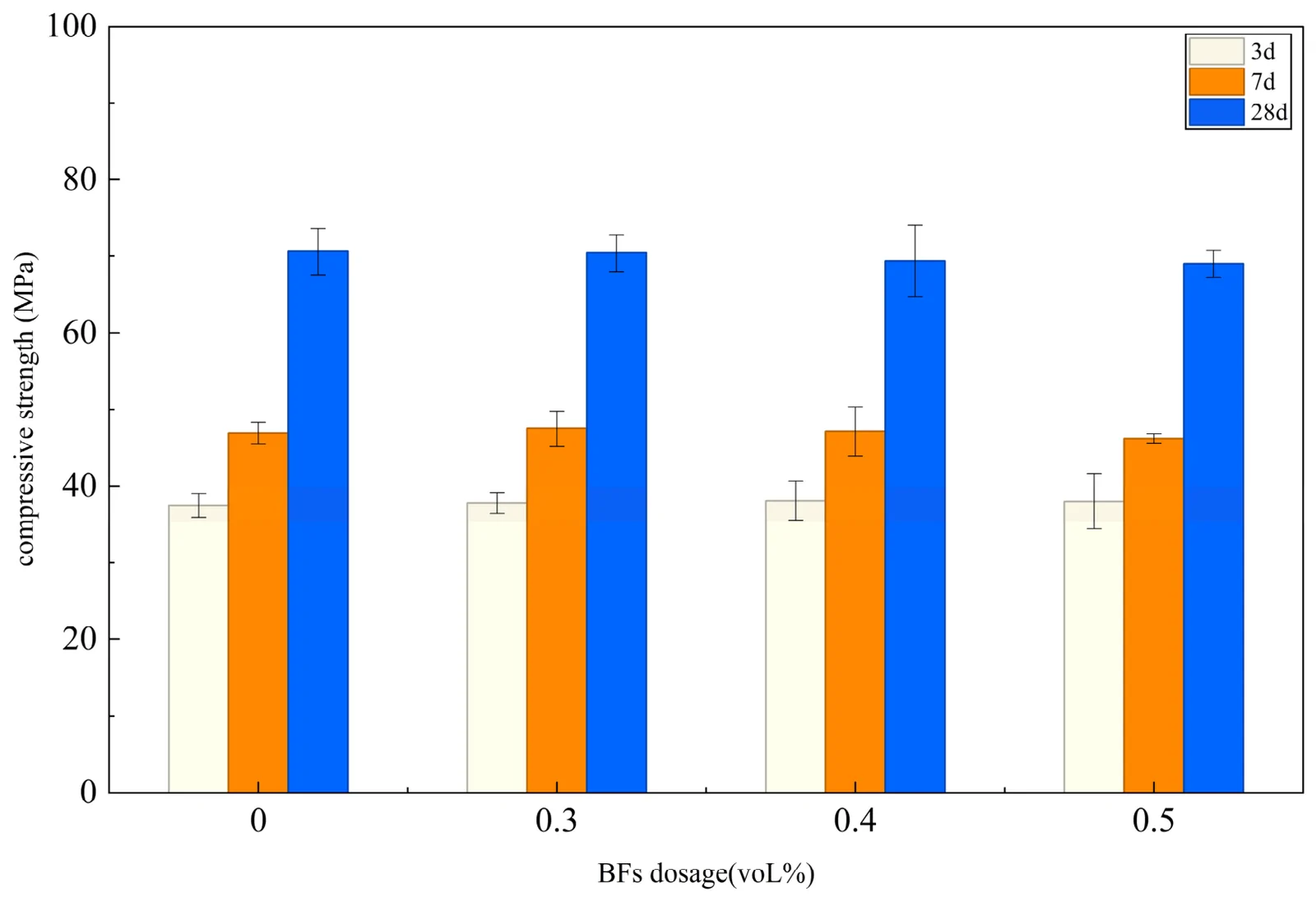
Effect of basalt fiber content on compressive strength. Error bars represent the standard deviations of valid replicate specimens.

**Figure 7:**
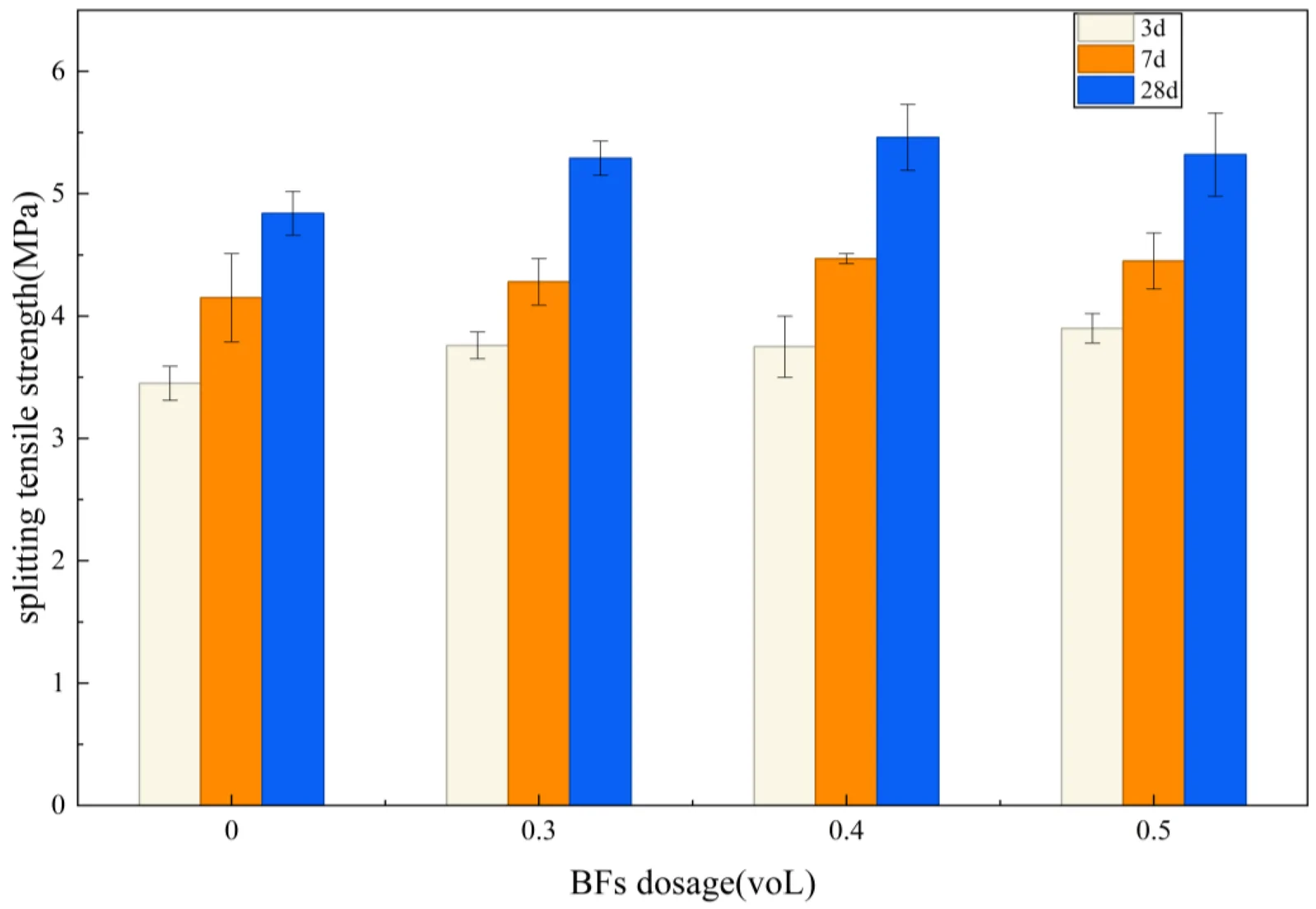
Effect of basalt fiber content on splitting tensile strength. Error bars represent the standard deviations of three replicate specimens.

To analyze the effect of basalt fiber content on the compression performance of desert sand high-strength concrete, three fiber content levels of 0.3%, 0.4% and 0.5% were set based on the DSHC-JZ reference mix proportion, and the compressive strength and splitting tensile strength at different ages were tested. The results are shown in Figure 6 and Figure 7, respectively.

As illustrated in Figure 6, the compressive strength values are presented as mean ± standard deviation, and the individual test results are provided in Table S1 in the Supplementary Materials. All mixtures exhibited a progressive increase in compressive strength with curing age, indicating that the matrix of desert sand high-strength concrete gradually densified with the development of hydration reactions. At 28 d, the compressive strength of the reference group DSHC-JZ was 70.6 MPa, while those of DSHC-BF0.3, DSHC-BF0.4 and DSHC-BF0.5 were 70.4, 69.5 and 69.0 MPa, respectively, corresponding to decreases of 0.28%, 1.56% and 2.27% compared with DSHC-JZ. This indicates that, within the fiber content range of 0.3–0.5%, basalt fiber had no obvious enhancement effect on peak compressive strength. This result is consistent with previous studies on basalt fiber-reinforced concrete [27,28], in which chopped fibers mainly improve tensile cracking resistance through bridging and crack-arresting effects, whereas compressive strength is more strongly governed by matrix compactness, the aggregate–paste interface and pore structure. With increasing fiber content, the difficulty of uniform fiber dispersion may increase, and local fiber clustering, interfacial pores or reduced workability may limit the beneficial crack-arresting effect, leading to a slight decrease in compressive strength [27].

In contrast to compressive strength, splitting tensile strength increased after basalt fiber incorporation. As shown in Figure 7, the splitting tensile strength values are presented as mean ± standard deviation, and the individual test results are provided in Table S2 in the Supplementary Materials. At 28 d, the splitting tensile strength of DSHC-JZ was 4.84 MPa. After incorporating 0.3%, 0.4% and 0.5% basalt fiber, the splitting tensile strengths increased to 5.29, 5.46 and 5.32 MPa, respectively, corresponding to increases of 9.30%, 12.81% and 9.92% compared with DSHC-JZ. Among them, DSHC-BF0.4 showed the highest splitting tensile strength, indicating the best tensile performance among the tested fiber contents. When the fiber content increased to 0.5%, the splitting tensile strength decreased slightly, which may be related to the increased difficulty of uniform fiber dispersion and possible local fiber clustering [27].

Based on the compressive and splitting tensile strength results, basalt fiber had a limited effect on compressive strength but a more pronounced effect on splitting tensile performance and crack resistance. DSHC-BF0.4 maintained relatively high compressive strength and achieved the highest 28 d splitting tensile strength. Therefore, DSHC-JZ, DSHC-BF0.4 and DSHC-BF0.5 were selected for the subsequent DIC crack evolution and microscopic mechanism analysis.

### 3.3. Variation of DIC Maximum Principal Strain During Compression Process

**Figure 8:**
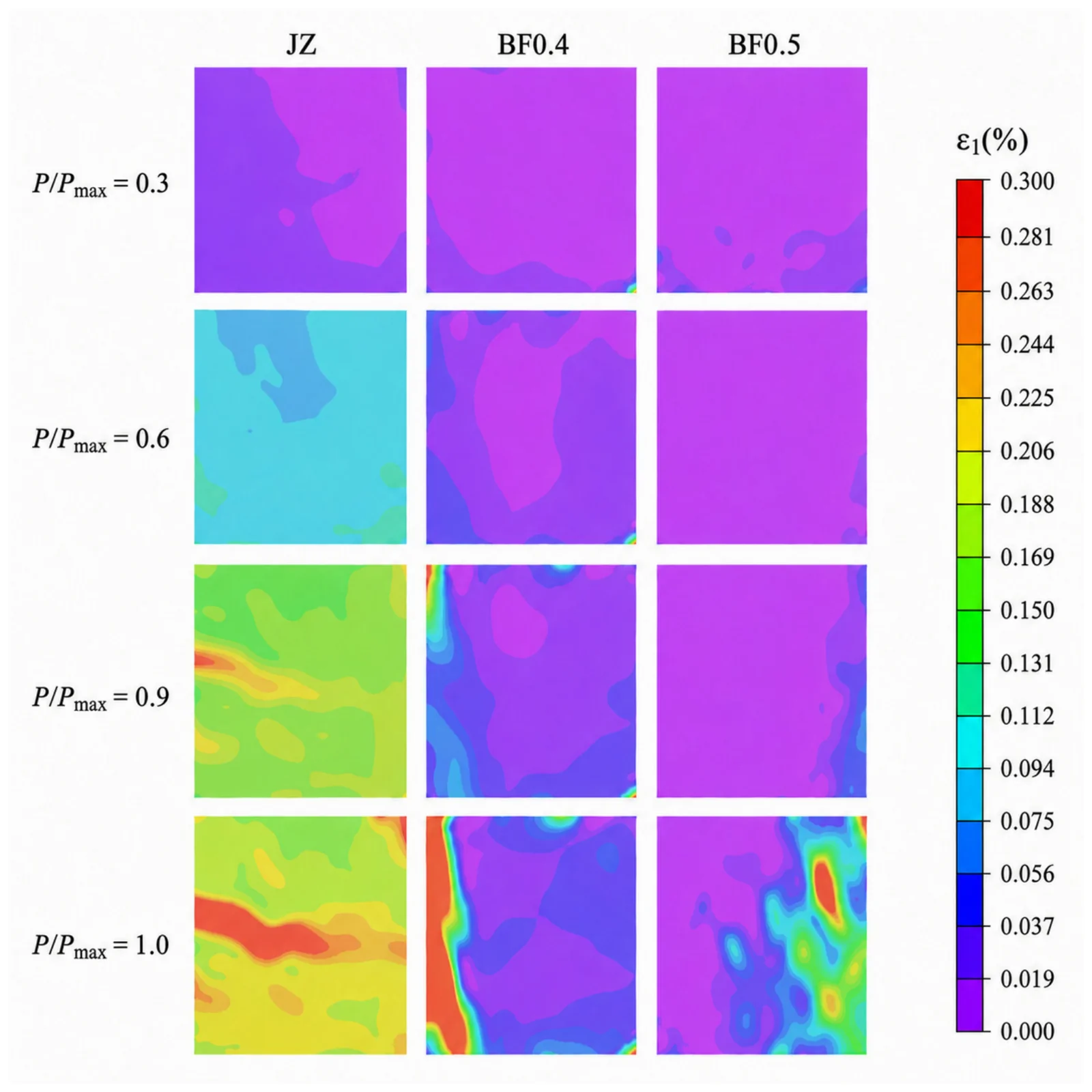
Evolution of maximum principal strain fields of representative DIC-monitored specimens with different basalt fiber contents during compression.

The DIC maximum principal strain field can intuitively reflect local deformation concentration and the process of crack initiation and propagation in concrete during compression, and is often used to analyze the transition of quasi-brittle materials from uniform deformation to localized failure [29,30]. To analyze the effect of basalt fiber on the compressive failure process of desert sand high-strength concrete, Figure 8 shows the maximum principal strain fields of representative DIC-monitored specimens from the DSHC-JZ, DSHC-BF0.4 and DSHC-BF0.5 groups at $P/P_{max}=0.3$, 0.6, 0.9 and 1.0.

As shown in Figure 8, the maximum principal strain increased with the load level from $0.3P_{max}$ to $1.0P_{max}$, and the strain field gradually changed from scattered deformation to localized concentration. In the DSHC-JZ specimen, a distinct high-strain zone appeared at the high-load stage, indicating obvious localized failure. Compared with DSHC-JZ, the DSHC-BF0.4 specimen showed a broader and more dispersed high-strain region, suggesting that a suitable basalt fiber content helped redistribute strain and delay the formation of a dominant localized failure zone [31]. For the DSHC-BF0.5 specimen, the high-strain region remained concentrated near the peak load, indicating that further increasing the fiber content did not lead to better deformation compatibility. This may reflect local differences in fiber distribution or interfacial condition. The representative DIC results suggest that DSHC-BF0.4 exhibited more dispersed strain localization under compression.

### 3.4. Evolution of DIC Maximum Principal Strain During Splitting Tensile Process

**Figure 9:**
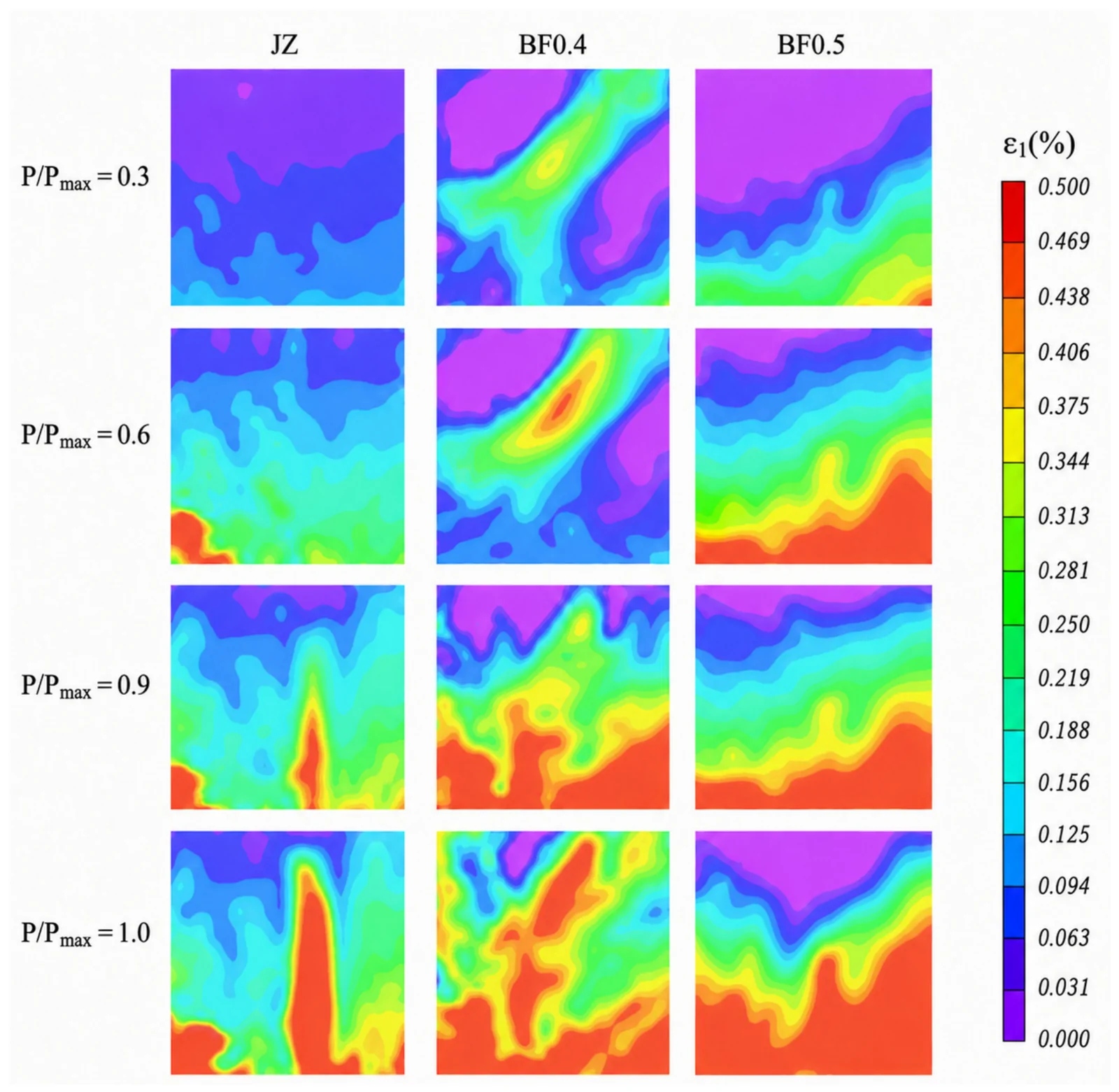
Evolution of maximum principal strain fields of representative DIC-monitored specimens with different basalt fiber contents during splitting tensile loading.

To quantify strain localization during splitting tensile loading, profile-line analysis was performed on representative DIC-monitored specimens of DSHC-JZ, DSHC-BF0.4 and DSHC-BF0.5. The strain fields shown in Figure 9 were obtained from individual representative specimens rather than averaged replicate results. The analysis was conducted at the peak-load stage $\left(P/P_{max}=1.0\right)$, when the strain-localization band was fully developed. Three horizontal profile lines were arranged at y/H=0.25, 0.50 and 0.75, passing through the vertical high-strain band along the loading centerline. Since splitting tensile failure is mainly controlled by tensile strain localization, only positive maximum principal strain values along each profile line were used. The peak and average positive maximum principal strains were extracted to calculate the strain concentration coefficient [31]. This coefficient reflects the degree of local tensile strain concentration along the selected profile line and is calculated according to Equation (3):

(3) $$K_{\varepsilon }=\frac{\varepsilon_{1,max}^{L}}{{\bar{\varepsilon }}_{1}^{L}}$$

where $K_{\varepsilon }$ is the strain concentration coefficient, $\varepsilon_{1,max}^{L}$ is the peak positive maximum principal strain along the selected profile line, and ${\bar{\varepsilon }}_{1}^{L}$ is the average positive maximum principal strain along the same profile line. A larger $K_{\varepsilon }$ indicates stronger local tensile strain concentration.

Figure 9 indicates that during the splitting tensile process, all groups of specimens form vertical high-strain bands along the loading centerline, indicating that the main crack mainly propagates along the splitting direction. Compared with DSHC-JZ, the high-strain region of DSHC-BF0.4 was broader and less concentrated, suggesting more dispersed strain localization around the developing main crack. For DSHC-BF0.5, the local high-strain region remained relatively concentrated near peak load, indicating that increasing the fiber content from 0.4% to 0.5% did not provide a clear additional improvement in strain redistribution.

Further combining the statistical results of the full-field maximum principal strain, the strain concentration coefficients of the three groups of specimens were analyzed, with the corresponding results summarized in Table 9.

As shown in Table 9, the strain concentration coefficient varied among the three profile lines, indicating that the calculated $K_{\varepsilon }$ was affected by the selected profile position. Therefore, the values of $K_{\varepsilon }$ at y/H=0.75, 0.50 and 0.25 are reported separately, together with the mean value and standard deviation. The mean $K_{\varepsilon }$ of the DSHC-JZ specimen was 2.60, showing obvious strain localization during splitting tensile loading. After basalt fiber was added, the mean $K_{\varepsilon }$ decreased to 1.83 for DSHC-BF0.4 and 1.84 for DSHC-BF0.5. This indicates that basalt fiber reduced local strain concentration and promoted a more dispersed strain distribution around the main crack. The mean $K_{\varepsilon }$ values of DSHC-BF0.4 and DSHC-BF0.5 were very close, suggesting that increasing the fiber content from 0.4% to 0.5% did not further improve strain redistribution. When considered together with the splitting tensile strength results, DSHC-BF0.4 showed a more favorable balance between tensile performance and representative strain redistribution.

### 3.5. Evolution Analysis of Crack Opening Displacement

**Figure 10:**
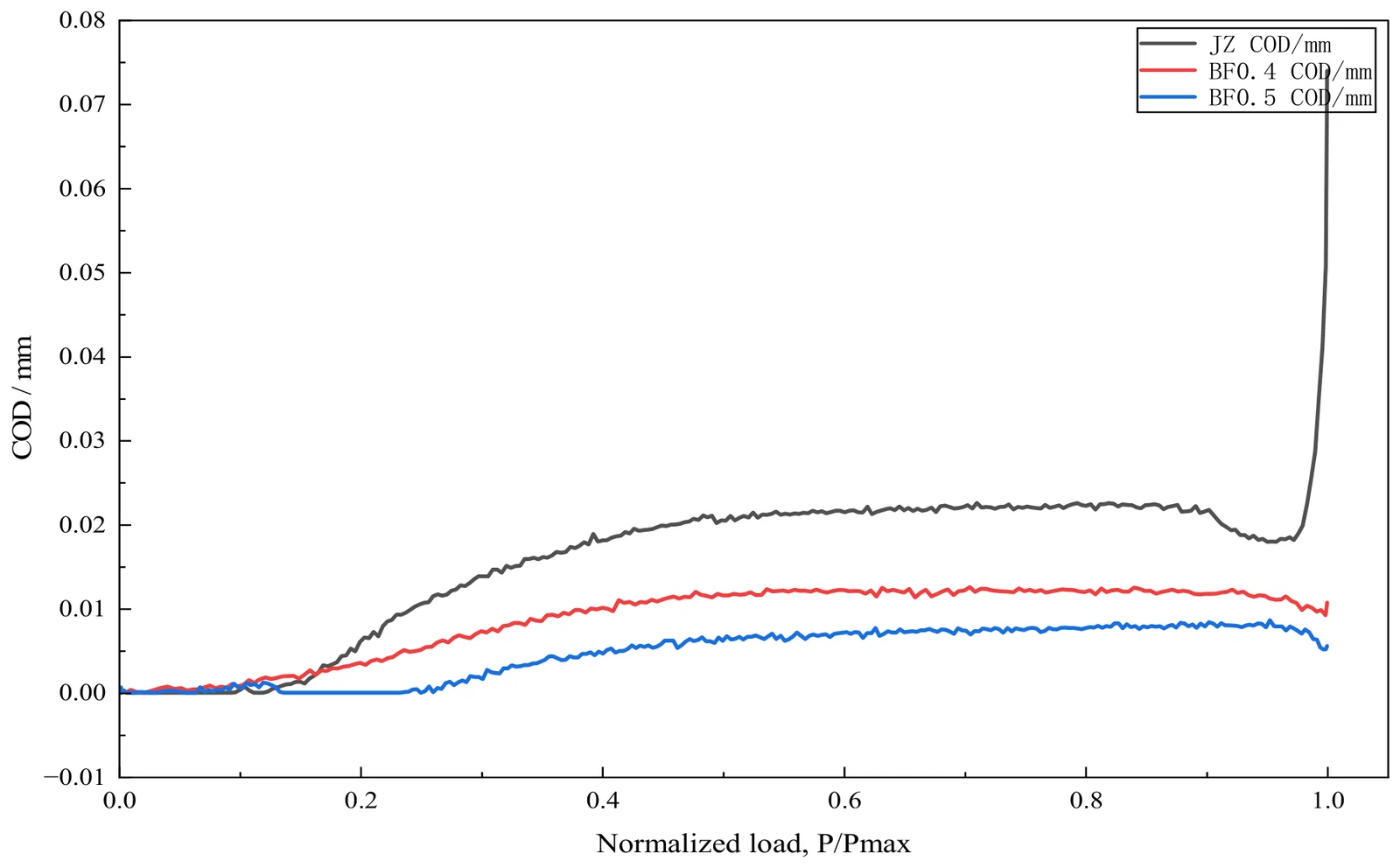
$P/P_{max}$–COD curves of representative DIC-monitored splitting tensile specimens with different basalt fiber contents.

To quantitatively evaluate crack opening during splitting tensile loading, COD curves were extracted from representative DIC-monitored specimens using virtual gauge-point pairs [31]. Three gauge pairs were arranged across the main crack at the upper, middle and lower positions, and their mean value was taken as the average COD of the specimen. Thus, the reported average COD represents a spatial average within the same specimen. The $P/P_{max}$-COD curves are shown in Figure 10.

Figure 10 shows that the COD increased with the normalized load $P/P_{max}$, indicating progressive opening of the main crack during splitting tensile loading. For DSHC-JZ, the COD increased rapidly near the peak-load stage, and the mean COD at peak load reached 0.074 mm, showing a more concentrated crack-opening process and a brittle splitting failure feature.

After basalt fiber incorporation, the COD was clearly reduced. The mean COD at peak load decreased to 0.011 mm for DSHC-BF0.4 and 0.006 mm for DSHC-BF0.5, while the corresponding maximum mean COD values were 0.013 mm and 0.009 mm, respectively. This indicates that basalt fiber restrained the opening of the main crack. The lower COD in the fiber-reinforced specimens is consistent with the crack-bridging features observed in SEM and with previous studies on fiber-reinforced cementitious composites [32].

Among the DIC-monitored specimens, DSHC-BF0.5 showed the lowest COD, indicating stronger restriction of crack opening. However, COD only reflects the crack-opening displacement at the selected virtual gauge positions and should be interpreted together with strain localization and splitting tensile strength.

The COD results obtained from fixed virtual gauge-point pairs are summarized in Table 10. Here, the COD at peak load refers to the value at $P/P_{max}=1.0$, and the maximum COD denotes the largest local COD recorded during the whole loading process. The mean COD was calculated from the gauge-point pairs at y/H=0.75, 0.50 and 0.25 within the same representative specimen.

As shown in Table 10, the mean COD at peak load decreased from 0.074 mm for DSHC-JZ to 0.011 mm and 0.006 mm for DSHC-BF0.4 and DSHC-BF0.5, respectively, corresponding to reductions of about 85% and 92%. The maximum mean COD values of the two fiber-reinforced groups were also much lower than that of DSHC-JZ, indicating that basalt fiber effectively restricted crack opening during splitting tensile loading.

For the fiber-reinforced specimens, the maximum mean COD was slightly higher than the COD at peak load, suggesting that the local opening at the fixed gauge positions may have reached its maximum before peak load. This phenomenon may be related to crack-path migration, crack branching or a shift of the dominant opening position, rather than actual crack closure.

Although DSHC-BF0.5 showed the smallest COD, its strain concentration coefficient was close to that of DSHC-BF0.4, indicating no further improvement in strain redistribution. Considering COD, strain localization and splitting tensile strength together, DSHC-BF0.4 showed a more favorable overall balance in the present system.

### 3.6. Microstructure and Reinforcement Mechanism Analysis

To clarify how basalt fiber influences crack initiation and propagation in high-strength concrete incorporating desert sand, this paper analyzes the microstructure and reinforcement mechanism of specimens with different fiber contents based on the SEM, XRD, TG-DTG and FTIR test results. The microscopic tests are mainly used to explain the aforementioned mechanical properties and DIC crack evolution results, with emphasis on analyzing whether the reinforcement effect of basalt fiber originates from changes in the hydration products of the matrix or from physical crack-arresting behavior, including fiber bridging and fiber–matrix interaction.

#### 3.6.1. SEM Micromorphology Analysis

**Figure 11:**
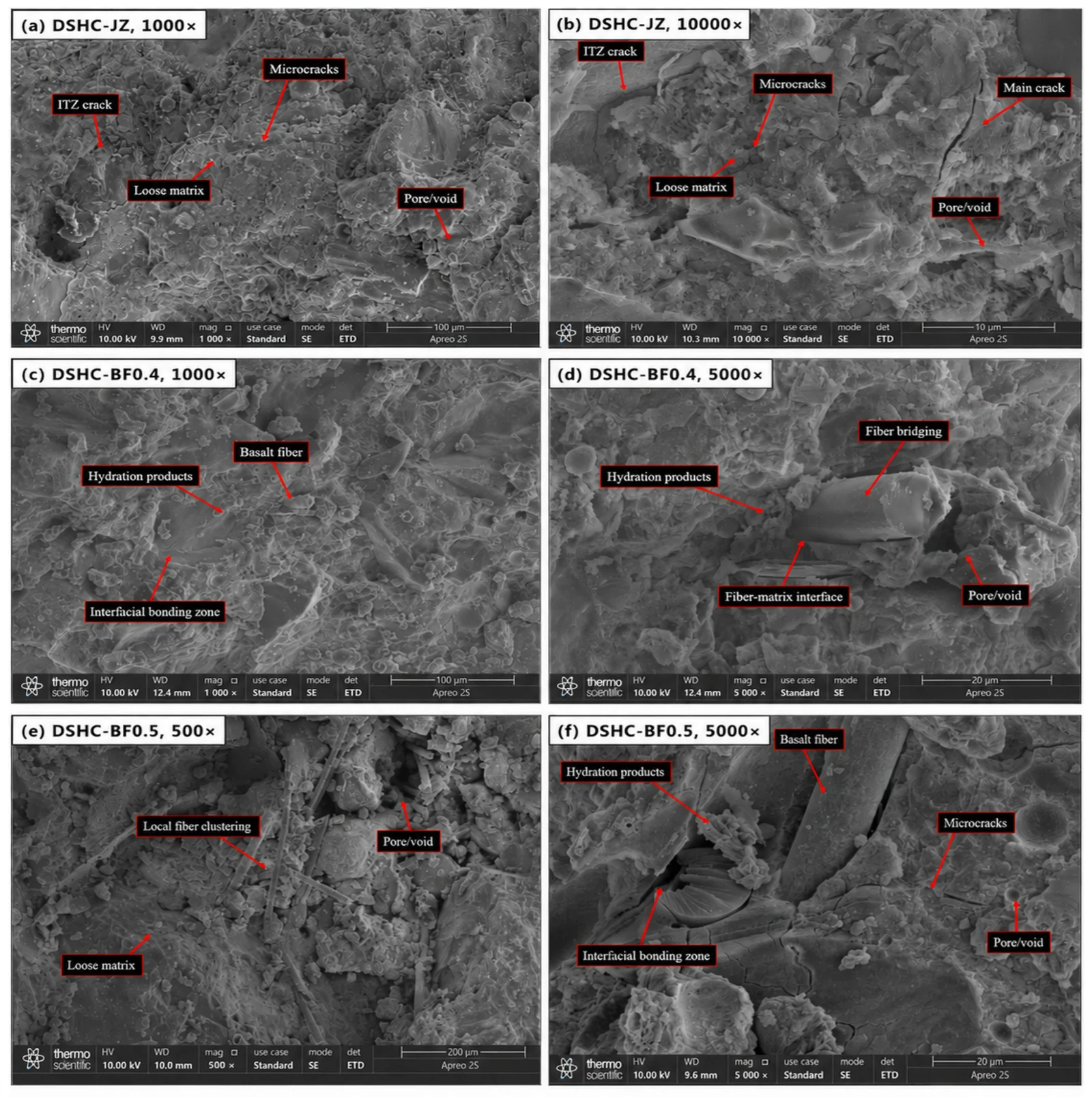
SEM images of representative fracture surfaces of DSHC-JZ, DSHC-BF0.4 and DSHC-BF0.5 at different observation areas and magnifications.

To explain the physical crack-arresting effect of basalt fiber from the perspectives of fracture morphology, fiber–matrix interface and crack propagation path, SEM observation was conducted on the fracture surfaces of typical specimens, and the results are shown in Figure 11.

Figure 11 shows SEM images of representative fracture surfaces of DSHC-JZ, DSHC-BF0.4 and DSHC-BF0.5 at different observation areas and magnifications. Figure 11a,b, Figure 11c,d and Figure 11e,f correspond to DSHC-JZ, DSHC-BF0.4 and DSHC-BF0.5, respectively.

As shown in Figure 11a,b, the DSHC-JZ specimen contains ITZ cracks, microcracks, pores/voids and loose matrix. The main crack propagates along weak matrix regions or the interfacial transition zone, and local pores and microcracks tend to connect with each other. This indicates that the reference specimen lacks effective cross-crack restraint, resulting in a relatively direct crack path and brittle fracture behavior.

Figure 11c,d shows the microstructure of DSHC-BF0.4. Basalt fibers are embedded in the hardened matrix, with hydration products attached around the fibers and a visible fiber–matrix interfacial zone. Fiber bridging, hydration products, pores/voids and the fiber–matrix interface can be observed at higher magnification. These features suggest that basalt fibers may provide crack-bridging restraint near the fracture region, which is consistent with the reduced COD and strain localization observed in the representative DIC results.

Figure 11e,f shows the SEM features of DSHC-BF0.5 at different magnifications. In Figure 11e, several basalt fibers are locally concentrated, with pores/voids observed in the surrounding matrix. At higher magnification, Figure 11f shows basalt fibers, hydration products, microcracks, pores/voids and the fiber–matrix interface. These local observations suggest that increasing the fiber content to 0.5% may make uniform fiber dispersion more difficult, which may partly explain the limited further improvement in tensile strength and strain redistribution.

Overall, the DSHC-JZ specimen showed relatively direct crack propagation through weak matrix regions, whereas the fiber-reinforced specimens exhibited visible fiber-bridging features and fiber–matrix interaction near the fracture region. The microstructural features observed in DSHC-BF0.4 were consistent with its higher splitting tensile strength and lower strain concentration in the representative DIC results.

#### 3.6.2. XRD Analysis

**Figure 12:**
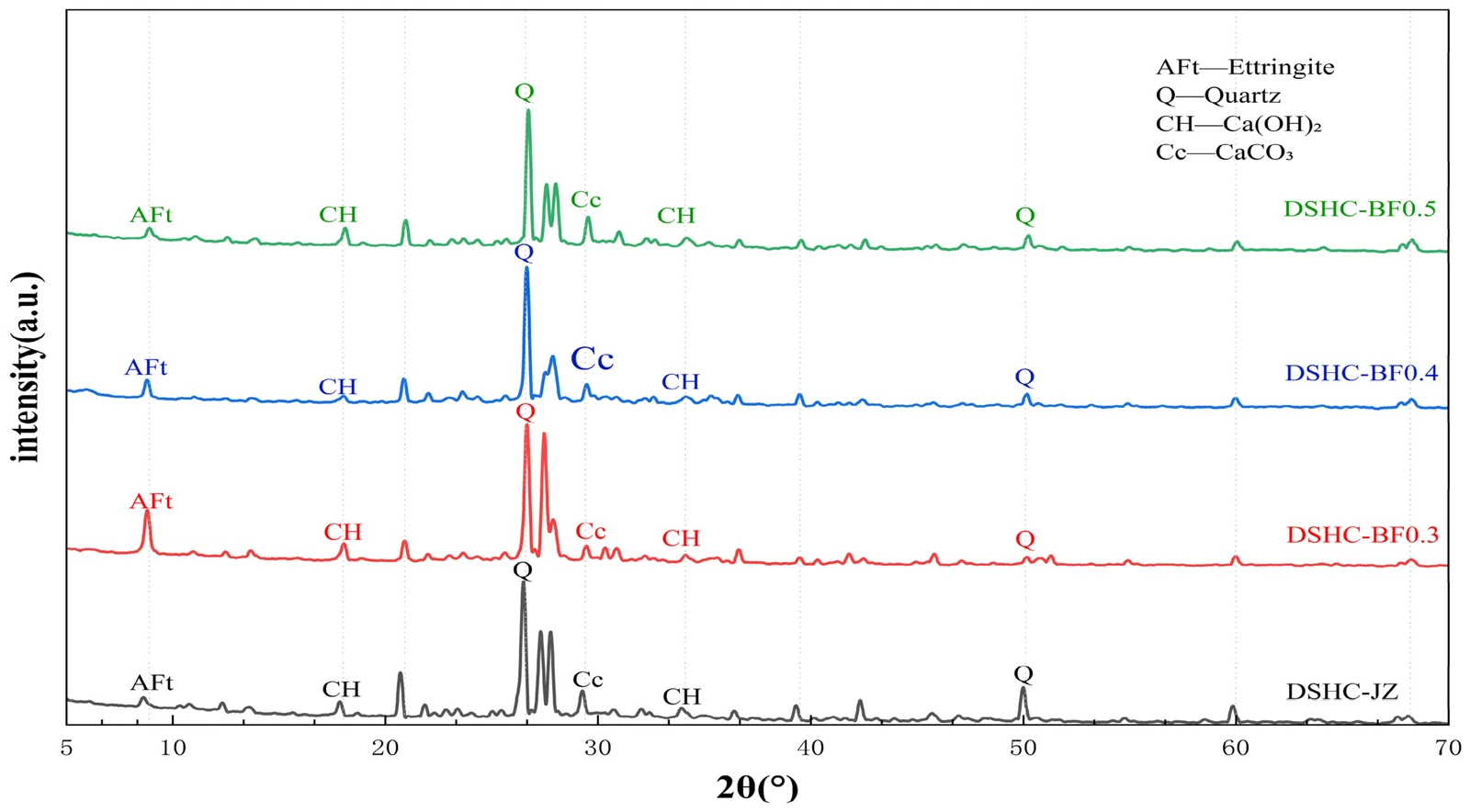
XRD patterns of specimens with different basalt fiber contents.

XRD was conducted on specimens with different fiber contents to examine whether basalt fiber incorporation altered the main crystalline phases of the hardened matrix. The XRD patterns are presented in Figure 12.

Figure 12 indicates that the positions of the main diffraction peaks of all groups of specimens are basically consistent, and the main crystalline phases include quartz (Q), Ca (OH)_2_ (CH), CaCO_3_ (Cc) and a small amount of AFt. Among them, the quartz peaks mainly originate from the siliceous components in the desert sand; CH and CaCO_3_ are related to cement hydration products and carbonate-type products, respectively. No obvious new diffraction peaks or clear peak-position shifts were observed among the groups with different fiber contents. This qualitative observation suggests that basalt fiber did not introduce new detectable crystalline phases in the hardened matrix [33].

It should be pointed out that C-S-H gel has relatively low crystallinity and usually appears as a broad diffuse background rather than sharp diffraction peaks in XRD patterns; therefore, XRD alone is not suitable for quantitative evaluation of C-S-H formation. Combined with the mechanical properties and representative DIC results, the XRD results suggest that, within the resolution of qualitative XRD observation, the performance improvement of DSHC-BF0.4 was not associated with the formation of obvious new crystalline phases.

#### 3.6.3. TG-DTG Analysis

**Figure 13:**
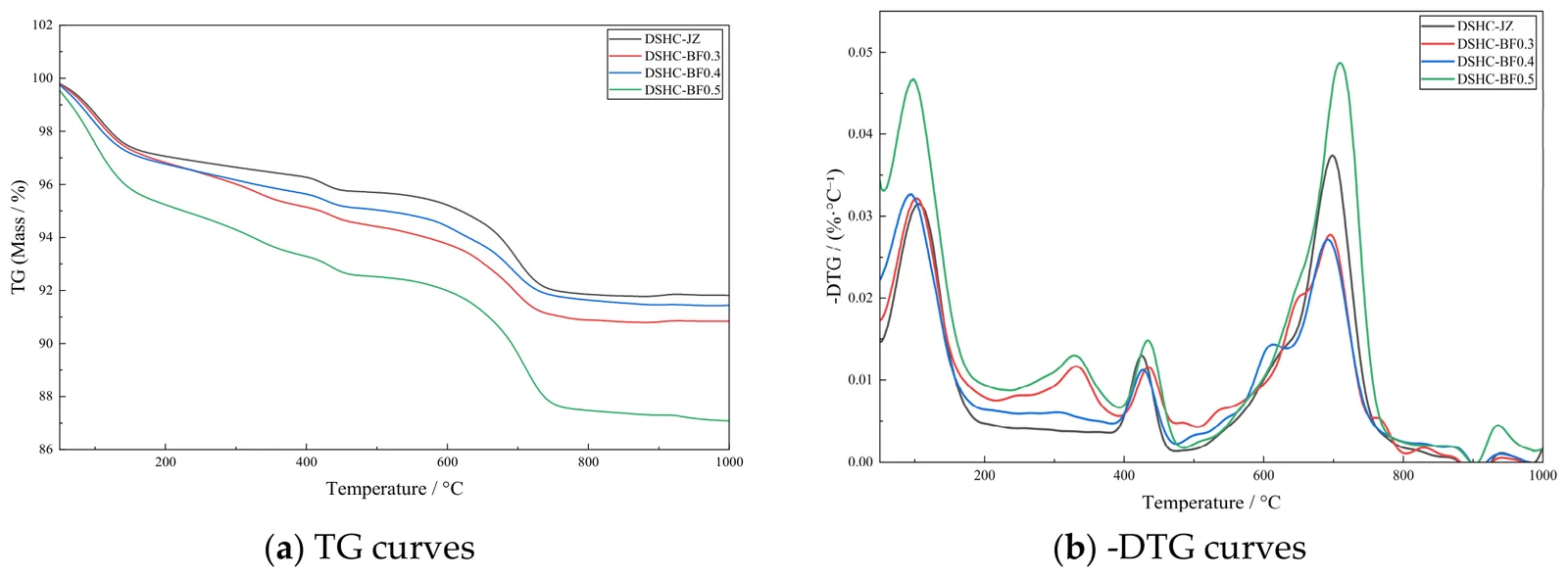
TG-DTG curves of specimens with different basalt fiber contents.

TG-DTG was used to compare the thermal decomposition behavior of the hardened matrix among mixtures with different fiber contents. The corresponding TG-DTG curves are shown in Figure 13.

Figure 13a shows that the TG-DTG curves of all groups present similar variation patterns. The main mass loss can be divided into three stages: the first stage occurs at approximately 50–300 °C, mainly corresponding to the removal of free water, adsorbed water and bound water in hydration products such as C-S-H gel and AFt; the second stage occurs at approximately 400–600 °C, mainly associated with the decomposition of Ca(OH)_2_; and the third stage occurs at approximately 600–800 °C, mainly corresponding to the decomposition of CaCO_3_ [33]. No obvious differences in the main decomposition temperature ranges were observed among the mixtures. Therefore, the TG-DTG results were used as qualitative support for comparing the hydration-related thermal behavior of different mixtures.

#### 3.6.4. FTIR Analysis

**Figure 14:**
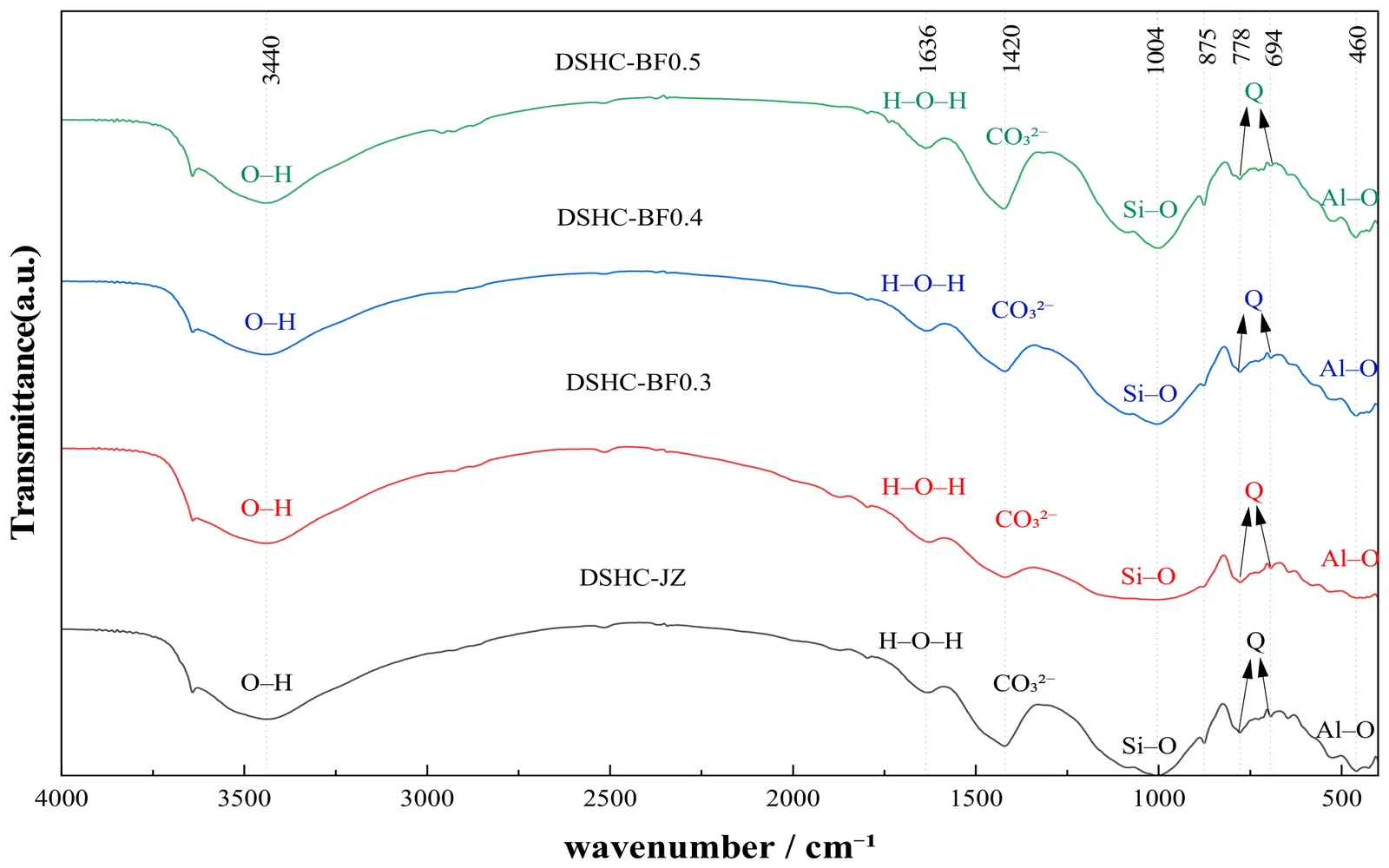
FTIR spectra of specimens with different basalt fiber contents.

FTIR was used to compare the main functional groups of mixtures with different fiber contents and complement the XRD and TG-DTG observations. The corresponding spectra are shown in Figure 14.

Figure 14 shows that the main absorption bands of all groups are generally similar. The broad band near 3400 cm^−1^ is related to O–H stretching vibration, and the band near 1640 cm^−1^ is attributed to H–O–H bending vibration. The bands near 1400–1500 cm^−1^ and 870 cm^−1^ are associated with CO_3_^2−^, while the band near 950–1100 cm^−1^ is mainly related to Si–O vibration in C–S–H gel or aluminosilicate structures. In addition, the bands near 780 cm^−1^ and 690 cm^−1^ are mainly related to Si–O bond vibration in quartz [34,35].

Compared with the reference group, the fiber-reinforced groups showed no obvious new absorption bands or differences in the main absorption-band positions. Therefore, the FTIR results were used as qualitative support for comparing the functional-group features of different mixtures.

Taken together, no obvious differences were observed in the detectable crystalline phases, main thermal decomposition stages or major FTIR absorption bands among the mixtures [33,34,35]. Combined with the SEM observations, these results suggest that the improved crack-control behavior was mainly associated with physical fiber bridging and fiber–matrix interaction rather than obvious chemical modification of the hardened matrix [36,37,38].

### 3.7. Discussion

Basalt fiber had a limited effect on compressive strength but a more pronounced influence on splitting tensile behavior and crack control. This agrees with previous studies showing that basalt fiber is generally more effective in improving tensile resistance, toughness and crack resistance than peak compressive strength [12,13,15,16]. The difference is mainly related to the failure mechanism. Compressive strength depends largely on matrix compactness, aggregate skeleton stability, pore structure and the aggregate–paste interface, whereas splitting tensile failure is governed by crack initiation and propagation [21,22,39]. During splitting, discrete fibers bridge developing cracks and restrain crack opening. The SEM observations in this study provide qualitative support for this crack-bridging behavior and fiber–matrix interaction [32,36,37,38].

The desert sand-replaced matrix may also influence fiber–matrix interaction. Its fine particle size and low fineness modulus can improve particle packing and fill local voids [6], potentially increasing contact between basalt fibers and the surrounding paste. Calcareous phases identified by XRD may also contribute to local matrix densification and fiber anchorage [25,32,36,37,38], whereas the rounded and relatively smooth desert sand particles may provide less mechanical interlocking than angular river sand. Thus, the fiber-reinforcing effect may result from the combined effects of particle filling, matrix densification and local anchorage, although interfacial bond-slip and pull-out behavior were not directly evaluated in this study.

The representative DIC and microscopic observations were also consistent with the overall crack-control interpretation. The representative DIC results showed reduced strain localization and smaller crack opening in the fiber-reinforced specimens, which agrees with previous DIC-based studies on crack evolution in concrete and fiber-reinforced cementitious materials [29,30,31,40]. SEM observations showed fiber bridging and fiber–matrix interaction near the crack region, supporting the physical crack-bridging interpretation [32,36,37,38]. Meanwhile, XRD, TG-DTG and FTIR showed no obvious changes in the main phases, thermal decomposition behavior or functional groups [33,34,35]. Thus, the improvement was mainly related to physical crack-bridging behavior and fiber–matrix interaction, rather than obvious chemical modification of the hardened matrix.

Increasing fiber content did not necessarily improve crack-control performance. Although DSHC-BF0.5 showed a smaller COD, it did not further improve strain redistribution or splitting tensile strength compared with DSHC-BF0.4. Since the statistical differences among the fiber-reinforced groups were not significant, 0.4% basalt fiber was regarded as the preferred dosage based on the overall performance in the present system.

## 4. Conclusions

(1) The orthogonal test results showed that the desert sand replacement ratio had the greatest influence on the comprehensive performance of desert sand high-strength concrete, followed by the water-to-binder ratio, while the fly ash content had a smaller effect. Based on the comprehensive scoring and range analysis, the mixture with a water-to-binder ratio of 0.32, a desert sand replacement ratio of 40% and a fly ash content of 20% was identified as the predicted preferred mixture. After confirmation testing, this mixture was used as the reference matrix, DSHC-JZ.
(2) Basalt fiber had limited influence on compressive strength, but increased splitting tensile strength. At 28 d, DSHC-BF0.4 reached the highest mean splitting tensile strength of 5.46 MPa, which was 12.81% higher than that of DSHC-JZ. When the fiber content increased to 0.5%, the splitting tensile strength decreased slightly, indicating that further increasing the fiber dosage did not provide additional tensile-strength improvement.
(3) DIC results showed that basalt fiber reduced strain localization and restricted crack opening during splitting tensile loading. The mean strain concentration coefficients of DSHC-BF0.4 and DSHC-BF0.5 were close, and both were lower than that of DSHC-JZ. Although DSHC-BF0.5 showed a smaller COD, its strain concentration coefficient was not further reduced and its splitting tensile strength did not exceed that of DSHC-BF0.4. Therefore, DSHC-BF0.4 achieved a better balance among crack-opening restriction, strain redistribution and tensile performance.
(4) No obvious differences were observed in the main detectable phases, thermal decomposition stages or functional groups among the mixtures. The observed performance improvement was mainly associated with fiber bridging and fiber–matrix interaction near the fracture region, rather than obvious chemical modification of the matrix. Considering the mechanical, DIC and microstructural results together, 0.4% basalt fiber was regarded as the preferred dosage in the present system.

## Figures and Tables

**Table 1:** Main chemical composition of desert sand by XRF.

Materials	SiO_2_/%	CaO/%	Al_2_O_3_/%	MgO/%	Fe_2_O_3_/%	K_2_O/%	Na_2_O/%	TiO_2_/%	P_2_O_5_/%	Cl/%	SO_3_/%
Desert sand	50.35	25.28	11.56	3.43	2.97	2.33	1.94	1.1	0.44	0.2	0.19

**Table 2:** Physical properties of river sand and desert sand.

Fine Aggregate	Apparent Density/kg·m^−3^	Bulk Density/kg·m^−3^	Fineness Modulus	Water Absorption/%
River sand	2635	1557.6	2.54	1.80
Desert sand	2625	1614.0	0.214	0.44

**Table 3:** Mix proportions of L9(3^3^) orthogonal test.

Group	Water-to-Binder Ratio	Desert Sand Replacement Rate/%	Fly Ash Content/%	Material Quantity/(kg/m^3^)						
				Cement	Fly Ash	Water	Desert Sand	Sand	Coarse Aggregate	Superplasticizer
DSHC1	0.32	30	0	520	0	170	212	495	1062	2.6
DSHC2	0.32	40	10	470	50	170	283	424	1062	2.6
DSHC3	0.32	50	20	420	100	170	354	354	1062	2.6
DSHC4	0.34	30	10	440	60	170	212	495	1062	2.5
DSHC5	0.34	40	20	400	100	170	283	424	1062	2.5
DSHC6	0.34	50	0	500	0	170	354	354	1062	2.5
DSHC7	0.36	30	20	370	100	170	212	495	1062	2.35
DSHC8	0.36	40	0	470	0	170	283	424	1062	2.35
DSHC9	0.36	50	10	420	50	170	354	354	1062	2.35

**Table 4:** Complete mix proportions of basalt fiber-reinforced DSHC mixtures.

Group	w/b	Cement /kg·m^−3^	Fly Ash /kg·m^−3^	Water /kg·m^−3^	Desert Sand /kg·m^−3^	River Sand /kg·m^−3^	Coarse Aggregate /kg·m^−3^	BF /kg·m^−3^	SP /% of binder	SP /kg·m^−3^	Slump /mm
DSHC-JZ	0.32	420	100	170	283	424	1062	0	0.50	2.60	184
DSHC-BF0.3	0.32	420	100	170	283	424	1062	8.07	0.70	3.64	172
DSHC-BF0.4	0.32	420	100	170	283	424	1062	10.76	0.90	4.68	168
DSHC-BF0.5	0.32	420	100	170	283	424	1062	13.45	1.10	5.72	161

Note: BF = basalt fiber; SP = superplasticizer. The basalt fiber mass was calculated from the fiber volume fraction and the fiber density of 2.69 g/cm^3^. The SP dosage is expressed as a percentage of the total binder mass.

**Table 5:** Main image acquisition and DIC calculation parameters.

Parameter	Value
Image resolution	5120 × 5120 pixels
Calibrated physical length	85 mm
Physical scale	0.0166 mm/pixel
Image acquisition interval	400 ms/frame
Subset size	80 × 80 pixels
Step size	80 pixels in both x- and y-directions
Strain-computation window	Half-window size of 2, corresponding to 5 × 5 calculation points
Filtering method	Gaussian polynomial smoothing
Correlation criterion	NCC, normalized cross-correlation
Treatment of out-of-plane motion	2D DIC; not explicitly corrected; minimized by perpendicular camera alignment and fixed camera-specimen configuration

**Table 6:** Comprehensive scores of L9(3^3^) orthogonal tests.

Group	Slump/mm	28 d Compressive Strength/MPa	28 d Splitting Tensile Strength/MPa	Comprehensive Score	Ranking
DSHC-1	156	67.63	3.76	0.901	3
DSHC-2	161	70.3	4.1	0.953	1
DSHC-3	155	65.5	3.42	0.858	8
DSHC-4	187	60.8	3.53	0.868	7
DSHC-5	182	69.5	3.72	0.934	2
DSHC-6	163	62.77	3.71	0.873	6
DSHC-7	211	62.03	3.34	0.882	5
DSHC-8	190	62.2	3.66	0.89	4
DSHC-9	186	59.6	3.45	0.852	9

**Table 7:** Range analysis of comprehensive scores.

Factor	k1	k2	k3	R	Order of Influence
A: Water-to-binder ratio	0.9040	0.8917	0.8747	0.0293	2
B: Desert sand replacement rate	0.8837	0.9257	0.8610	0.0647	1
C: Fly ash content	0.8880	0.8910	0.8913	0.0033	3

**Table 8:** Comparison between the best directly tested mixture and the A1B2C3 confirmation mixture.

Mixture	w/b	Desert Sand Replacement/%	Fly Ash Content/%	Slump/mm	28 d Compressive Strength/MPa	28 d Splitting Tensile Strength/MPa	Description
DSHC-2	0.32	40	10	161	70.30	4.10	Best directly tested mixture
DSHC-JZ	0.32	40	20	184	70.60	4.84	Confirmation mixture

**Table 9:** Line-based strain concentration coefficients at the peak-load stage (P/Pmax = 1.0).

Mixture	Profile Position (y/H)	$\varepsilon_{1,\max}^{L}$	${\bar{\varepsilon }}_{1}^{L}$	$K_{\varepsilon }$
DSHC-JZ	0.75	0.460	0.193	2.38
DSHC-JZ	0.50	0.897	0.304	2.95
DSHC-JZ	0.25	1.130	0.456	2.48
DSHC-JZ	Mean ± SD	—	—	2.60 ± 0.30
DSHC-BF0.4	0.75	0.234	0.106	2.20
DSHC-BF0.4	0.50	0.332	0.188	1.76
DSHC-BF0.4	0.25	0.418	0.276	1.52
DSHC-BF0.4	Mean ± SD	—	—	1.83 ± 0.35
DSHC-BF0.5	0.75	0.055	0.024	2.28
DSHC-BF0.5	0.50	0.155	0.086	1.81
DSHC-BF0.5	0.25	0.240	0.168	1.43
DSHC-BF0.5	Mean ± SD	—	—	1.84 ± 0.43

Note: The values were extracted from representative DIC-monitored splitting tensile specimens at the peak-load stage ($P/P_{max}$ = 1.0). y/H = 0.75, 0.50 and 0.25 correspond to the upper, middle and lower profile lines, respectively. Only positive maximum principal strain values along each profile line were used to calculate ${\bar{\varepsilon }}_{1}^{L}$ and $K_{\varepsilon }$. The standard deviation represents the spatial variation among the three profile lines within the same representative specimen, rather than the variation among replicate specimens.

**Table 10:** Characteristic COD parameters extracted from fixed virtual gauge-point pairs.

Mixture	Gauge Position $\left(y/H\right)$	COD at Peak Load/mm	Maximum COD/mm
DSHC-JZ	y/H = 0.75	0.007	0.007
DSHC-JZ	y/H = 0.50	0.087	0.087
DSHC-JZ	y/H = 0.25	0.128	0.128
DSHC-JZ	Mean	0.074	0.074
DSHC-BF0.4	y/H = 0.75	0.007	0.007
DSHC-BF0.4	y/H = 0.50	0.012	0.012
DSHC-BF0.4	y/H = 0.25	0.014	0.023
DSHC-BF0.4	Mean	0.011	0.013
DSHC-BF0.5	y/H = 0.75	0.004	0.006
DSHC-BF0.5	y/H = 0.50	0.004	0.005
DSHC-BF0.5	y/H = 0.25	0.009	0.015
DSHC-BF0.5	Mean	0.006	0.009

Note: COD was extracted from fixed virtual gauge pairs with an initial gauge length of $L_{g}=10$ mm. The gauge pairs were arranged across the main crack at y/H=0.75, 0.50 and 0.25, with each point located about 5 mm from the crack. “COD at peak load” denotes the value at $P/P_{max}=1.0$, while “Maximum COD” denotes the largest COD recorded during loading. The mean value was obtained by averaging the three gauge pairs within the same representative specimen.

## Data Availability

The original contributions presented in this study are included in the article/Supplementary Material. Further inquiries can be directed to the corresponding author.

## Supplementary Materials

The following supporting information can be downloaded at: https://www.mdpi.com/article/10.3390/ma19163486/s1, Table S1: Individual compressive strength results and statistical parameters; Table S2: Individual splitting tensile strength results and statistical parameters; Table S3: Significance test results for 28 d mechanical properties among different basalt fiber contents.
