# Supplementary material for: Study on Mechanical Properties and Crack Evolution of Basalt Fiber-Reinforced Desert Sand High-Strength Concrete Based on DIC
Source: Materials (Basel). 2026 Aug 18;19(16):3486. doi: 10.3390/ma19163486 (PMC13514799; doi:10.3390/ma19163486)
Supplement: Supplementary file 1 [file materials-19-03486-s001.zip › materials-4477758-supplementary.pdf]

Supplementary Materials

Table S1. Individual compressive strength results and statistical parameters.

Mixture	Age / d	Specimen 1 / MPa	Specimen 2 / MPa	Specimen 3 / MPa	n	Mean / MPa	SD / MPa
DSHC-JZ	3	36.00	39.10	37.40	3	37.50	1.55
DSHC-JZ	7	45.40	47.20	48.10	3	46.90	1.37
DSHC-JZ	28	67.30	73.20	71.30	3	70.60	3.01
DSHC-BF0.3	3	36.40	37.90	39.10	3	37.80	1.35
DSHC-BF0.3	7	44.90	49.10	48.50	3	47.50	2.27
DSHC-BF0.3	28	68.70	72.10	57.70*	2	70.40	2.40
DSHC-BF0.4	3	38.00	35.60	40.70	3	38.10	2.55
DSHC-BF0.4	7	45.50	45.00	50.80	3	47.10	3.21
DSHC-BF0.4	28	69.80	64.50	73.80	3	69.37	4.67
DSHC-BF0.5	3	41.60	34.40	38.00	3	38.00	3.60
DSHC-BF0.5	7	45.60	46.80	46.20	3	46.20	0.60
DSHC-BF0.5	28	70.20	67.80	46.00*	2	69.00	1.70

Note: Values marked with * were not included in the calculation of mean and standard deviation. SD represents the sample standard deviation calculated from valid replicate values.

Table S2. Individual splitting tensile strength results and statistical parameters.

Mixture	Age / d	Specimen 1 / MPa	Specimen 2 / MPa	Specimen 3 / MPa	n	Mean / MPa	SD / MPa
DSHC-JZ	3	3.32	3.44	3.59	3	3.45	0.14
DSHC-JZ	7	3.75	4.24	4.46	3	4.15	0.36
DSHC-JZ	28	4.70	4.78	5.04	3	4.84	0.18
DSHC-BF0.3	3	3.68	3.84	—	2	3.76	0.11
DSHC-BF0.3	7	4.21	4.49	4.14	3	4.28	0.19
DSHC-BF0.3	28	5.13	5.34	5.40	3	5.29	0.14
DSHC-BF0.4	3	3.52	4.02	3.71	3	3.75	0.25
DSHC-BF0.4	7	4.50	4.49	4.42	3	4.47	0.04
DSHC-BF0.4	28	5.23	5.39	5.76	3	5.46	0.27
DSHC-BF0.5	3	4.03	3.88	3.79	3	3.90	0.12
DSHC-BF0.5	7	4.72	4.30	4.33	3	4.45	0.23
DSHC-BF0.5	28	4.95	5.39	5.62	3	5.32	0.34

Note: "—" indicates that no valid value was available. SD represents the sample standard deviation calculated from valid replicate values.

Table S3. Significance test results for 28 d mechanical properties among different basalt fiber contents.

Property	Valid n (JZ/BF0.3/BF0.4/ BF0.5)	Overall Welch ANOVA	Pairwise comparison (Games–Howell)	p-value	Result
28 d compressive strength	3/2/3/2	$F(3, 3.12) = 0.185$; $p = 0.900$	DSHC-JZ vs DSHC-BF0.3	1.000	ns
			DSHC-JZ vs DSHC-BF0.4	0.978	ns
			DSHC-JZ vs DSHC-BF0.5	0.869	ns
			DSHC-BF0.3 vs DSHC-BF0.4	0.986	ns
			DSHC-BF0.3 vs DSHC-BF0.5	0.899	ns
			DSHC-BF0.4 vs DSHC-BF0.5	0.999	ns
28 d splitting tensile strength	3/3/3/3	$F(3, 4.26) = 4.181$; $p = 0.094$	DSHC-JZ vs DSHC-BF0.3	0.090	ns
			DSHC-JZ vs DSHC-BF0.4	0.111	ns
			DSHC-JZ vs DSHC-BF0.5	0.308	ns
			DSHC-BF0.3 vs DSHC-BF0.4	0.780	ns
			DSHC-BF0.3 vs DSHC-BF0.5	0.999	ns
			DSHC-BF0.4 vs DSHC-BF0.5	0.940	ns

Note: Welch ANOVA was used for the overall comparison because the valid sample sizes were small and unequal for some groups. Games–Howell post-hoc tests were used for pairwise comparisons. ns indicates no significant difference at $p \geq 0.05$. The 28 d compressive strength values marked with * in Table S1 were excluded from the analysis.

Interpretation: No significant differences were detected among the tested basalt fiber contents at $p < 0.05$. Therefore, the 0.4% basalt fiber group should be described as the preferred dosage based on comprehensive performance rather than as a statistically proven optimum.
